# Bird-building collision risk: An assessment of the collision risk of birds with buildings by phylogeny and behavior using two citizen-science datasets

**DOI:** 10.1371/journal.pone.0201558

**Published:** 2018-08-09

**Authors:** K. Samantha Nichols, Tania Homayoun, Joanna Eckles, Robert B. Blair

**Affiliations:** 1 Conservation Sciences Graduate Program, University of Minnesota, Saint Paul, MN, United States of America; 2 Audubon Minnesota, Saint Paul, MN, United States of America; 3 Department of Fisheries, Wildlife, and Conservation Biology, University of Minnesota, Saint Paul, MN, United States of America; Universidade de Lisboa Instituto Superior de Agronomia, PORTUGAL

## Abstract

Bird collisions with buildings are the second largest anthropogenic source of direct mortality for birds (365–988 million birds killed annually in the United States). Recent research suggests that this mortality occurs disproportionately across species. However, previous work had relied on regional and annual measures of relative species abundance. Our research identifies which species experience higher or lower collision rates than expected from local abundances using two sets of citizen science data: Minnesota Project BirdSafe and the Mississippi River Twin Cities Important Bird Area Landbird Monitoring Program. Our analysis used a measure of relative species abundance that spatially overlaps the area monitored for building collisions and was measured weekly, allowing for a temporally and spatially more specific analysis than most previous analyses. Abundance and collision data were used to model phylogenetic and behavioral traits associated with increased collision risk. Behavioral traits included diurnal/nocturnal migration timing, length of migration, and foraging strategies. Our analysis shows that birds that predominately migrate during the day have a decreased risk of building collisions despite peak collision numbers occurring during early morning; this result suggests that more nuanced behavioral or physiological differences between diurnal and nocturnal migrants could contribute to bird-building collision risk. Additionally, for many species, local abundance is the predominant determining factor for collision risk. However, for ~20% of species studied, the family, genus, and/or species of a bird may affect the collision risk.

## Introduction

Bird collisions with buildings cause a huge proportion of anthropogenic direct mortality to birds, second only to predation by cats [1]. The most recent national estimate of mortality from building collisions is 365–988 million birds killed annually in the United States [2]. With an estimated avian breeding population of 3.2 billion in the United States [3], these bird-building collision numbers correspond to an annual decrease of between 11% and 31% in the avian population, although it is important to note that there is very low precision in this estimated breeding population size as well as huge annual fluctuations in avian population size as part of the reproductive cycle. While it has been postulated that these deaths may be largely compensatory [4–6] it is undeniable that for species already at risk from a multitude of pressures, the deaths caused by building collisions only exacerbate the threats of population decline and even extinction [4,7].

The majority of existing research on bird-building collisions has focused on the buildings involved in these events. Initial research on bird-building collisions highlighted and described the conservation threat [8,9]. Later studies examined collisions in different habitats ranging from urban [4,10–14] to rural systems [10,15]. Some studies have compared the collisions at buildings in urban and rural areas [2,16,17]; in particular, while individual buildings in urban areas can have very high annual collision numbers, the sheer magnitude of non-urban buildings means that these individually smaller collision rates have an aggregately larger contribution to total bird-building collisions [2,18]. Research has also been done on the timing of collisions, including documenting peak collision frequencies in the early morning [9,11] and during spring and fall migration [10,12,13,19,20]. Much of the recent research has focused on how landscape factors, especially increased vegetation, [10–12,14,17,19–21] or building factors, especially increased glass area, [12,14,16,19,21] correlate with increased numbers of avian collisions.

Until recently, researchers have approached bird-building collisions with the primary focus on the buildings and the glass within those buildings rather than the birds that collide with those windows. The focus on buildings and not birds is largely due to an early publication that found building mortalities to be non-selective by species, age, sex, or condition of birds [9]. Though a fundamental paper, Klem relied on those birds marked as collision fatalities in a survey of existing museum collections and 2 years of surveys of a few buildings in Southern Illinois [9]. Klem’s paper was groundbreaking when published in terms of highlighting the need for further study of bird-building collisions as a conservation threat and a notable source of direct avian mortality. However, as one of the first paper’s published in this field, its scope and scale of replication were understandably limited [9]. Nevertheless, these conclusions were rarely revisited, even as citizen-science monitoring efforts exponentially increased the number of birds recorded as injured or killed by building collisions (however see [20,22]). Recently, some of these large datasets have been used to re-investigate the question of differential susceptibility to building collisions across species; in other words, testing if some species experience increased mortality from building collisions disproportionate to their abundance. The findings from these studies seem to refute Klem’s original conclusion; some species of birds appear to be substantially more susceptible to building collisions than expected by their population size (supercolliders) while other species appear much less likely to hit buildings than their population size would suggest (superavoiders) [2,4]. Additionally, Arnold and Zink found some factors that may explain increased susceptibility for some species, with nocturnal migrants, long distance migrants, and some phylogenetic groups being more susceptible to building collisions [4].

While these 2 previous studies of species susceptibility used different datasets of collisions, they both relied on aggregated Breeding Bird Survey (BBS) data to estimate the relative population sizes of each species in their studies, which is an imperfect means of determining relative species abundances [2,4]. BBS data provide an excellent large-scale perspective of species population trends and ranges across North America [23,24], and is an appropriate dataset to use when working with collision data from a broad geographic range as was the case for both the previous studies [2,4]. However, BBS does a poorer job of detecting small scale variability among species, as well as intra-annual variability within species [23,25]. While these large-scale bird-building studies have shown that differential species susceptibility exists, they are unable to capture or examine fine grain details, such as temporal or regional variability in susceptibility [2,4]. It is therefore valuable to assess the question of variability in susceptibility to collisions at a local scale to complement these larger scale studies. One previous study looks at species susceptibility at a local scale, but was only able to collect data on abundance and collisions for a single location with 6 buildings over one spring and one fall migration [26]. Our analysis seeks to further fill the knowledge gap of species susceptibility by using spatially restricted and temporally defined data on both bird-building collisions and community composition during spring migration across multiple years and numerous buildings to explore differential susceptibility to bird-building collisions.

In Minnesota, the data collected for 2 citizen-science monitoring programs make it possible to test for unequal species susceptibility to building collisions in comparison to locally collected measures of relative population sizes of migrating species. One project, Minnesota Project BirdSafe (hereafter Project BirdSafe), had volunteers monitor for dead and injured birds from building collisions along defined routes in downtown Minneapolis and St. Paul. These routes were walked daily during spring and fall migration seasons starting in the spring of 2007. The second project, the Mississippi River Twin Cities Important Bird Area Monitoring Program, had volunteers collect point count data at several locations along the Mississippi River in the greater metropolitan area from 2007 to 2010. Point counts were made on a weekly basis in the spring season and at least twice monthly during the summer season. From these 2 citizen-science monitoring programs, we have species-specific collision mortality records on a daily basis and an index for relative population sizes on a weekly basis, with temporal overlap for the spring migration seasons of 2007 through 2010. Thus we can test which species are most susceptible to building collisions in Minneapolis and St. Paul during spring migration using locally reliable population data with a temporal component.

In this paper, we test for differential susceptibility to bird-building collisions by 3 sets of criteria: {1} susceptibility by species, {2} susceptibility by higher-level phylogeny (e.g. family, genus), and {3} susceptibility by behavior (including migration and foraging). Using generalized linear mixed models (GLMM) with data from both Project BirdSafe (collision records) and Mississippi River Twin Cities Important Bird Area Landbird Monitoring Program (point count data), we test these 3 categories of susceptibility.

## Materials and methods

### BirdSafe dataset

**Fig 1:**
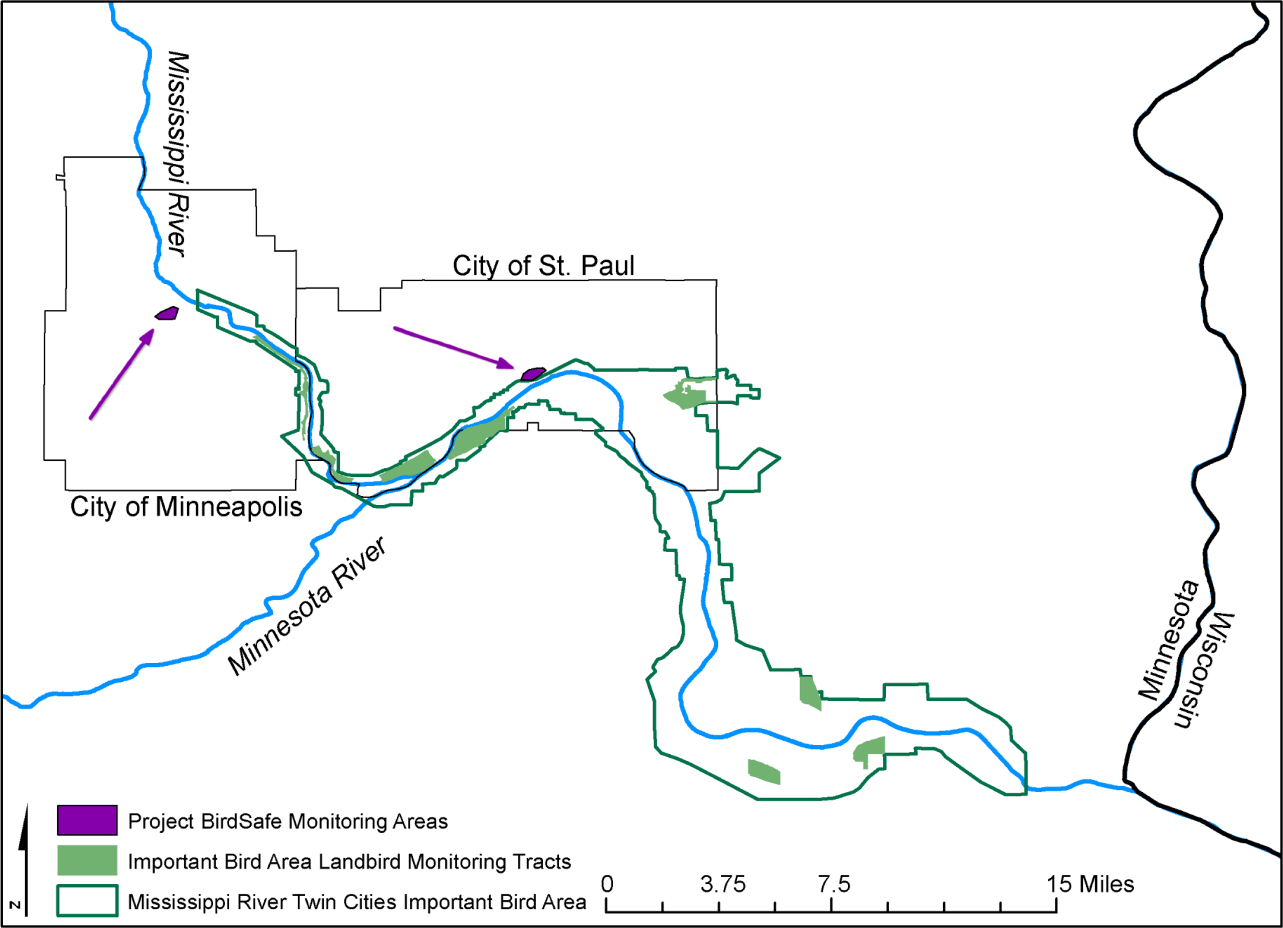
Map of study region showing relative positions of monitoring areas for project BirdSafe and Important Bird Area Landbird Monitoring Program. Map of the greater Minneapolis-St. Paul metro area in Minnesota, USA. Project BirdSafe monitoring areas are indicated by shaded purple regions. The Mississippi River Twin Cities Important Bird Area (IBA) is outlined in green and the sites within the IBA that were monitored as part of the Landbird Monitoring project are indicated with solid green.

Volunteers for Project BirdSafe patrolled 2 routes (one route for Minneapolis and one route for St. Paul) every morning, between 06:00 and 10:00, during spring and fall migrations (Fig 1; [13]). Each route was approximately 3 km long and together included buildings ranging from 2 to 57 stories and with a range of architectural styles including quantity of glass (building façades ranged from 0 to almost 100% glass). Monitoring dates were set based on local migration timing and went from mid-March through May (spring migration) and mid-August through October (fall migration). Though monitoring for this project began in spring of 2007 and continued through fall 2016, we used spring 2007 through spring 2010 data (S1 Table) for this analysis as this data range overlapped with the Important Bird Area (IBA) monitoring data; as all analyses started with comparing collisions to relative abundance, only collision data with a corresponding abundance measure could be included in analysis. All birds found were recorded by location (building and building side), date, and time, as well as species [13]. Live birds were released away from the downtown area or taken to the Wildlife Rehabilitation Center of Minnesota depending on condition of bird. Dead birds were collected and species was confirmed by expert examination. The carcasses were later processed for weight, genetic sex, age, wing length, fat condition, and stomach contents [13]. A spread wing and tissue sample from each dead bird collected are stored as part of the Bell Museum of Natural History’s collection [13]. Since the IBA data were not available for every day of the monitoring season, the BirdSafe accounts are also summed for each species by week (S7 Table).

Because monitoring routes were fixed, we had relatively constant searcher effort (by distance traveled, though not by time) and thus have meaningful zeros–that is, if a species is not recorded on a given day, we assumed it is because no individuals of that species were found rather than a lack of recording for that species. While there was likely imperfect detection of collisions along the route (because of searcher error, street sweepers, and scavenger effect, etc.), previous analysis by Arnold and Zink [4] found no effect of size or conspicuousness on apparent vulnerability, suggesting that any biases in detectability across species are relatively small. Therefore, we used the total number of collisions per species per week as an index of relative collision vulnerability over time.

An additional aspect of the Project BirdSafe monitoring was to encourage buildings to participate in a “lights out” effort, where lights in buildings were turned off during spring and fall migration. Of the approximately 100 buildings monitored by Project BirdSafe, 2 were engaged in “lights out” prior to the start of monitoring in spring of 2007, 8 joined during spring 2007, 1 joined during spring 2008, 4 joined during spring 2009, and 3 joined during spring of 2010. Thus less than 20% of all buildings were engaged in “lights out” programs during this study. It is likely that these changes affected collisions at individual buildings and the overall trend of increasing “lights out” buildings may have even contributed to an annual pattern in collisions, but was not the focus of this analysis and therefore the extent of “lights out” buildings was not incorporated into our analysis.

#### BirdSafe ethics statement

Bird carcasses were collected under Minnesota Department of Natural Resources salvage permits (Numbers 14827, 20412) and US Fish and Wildlife Federal Fish and Wildlife Permits (Numbers MB785154-0, MB54075B-0). Surveys were targeted for dead birds, and no live birds were restrained unless injured, and only then for transportation away from buildings and occasionally to veterinary care if necessary. Therefore, no protocols were submitted for IACUC or other animal care committee review; nevertheless, all citizen scientists were trained on protocols, including handling of injured live birds, to minimize stress and additional injury to birds.

### IBA monitoring dataset

Beginning in 2007, the Mississippi River Twin Cities Important Bird Area (IBA) was monitored for landbird abundance to determine how these birds used the IBA, particularly during migration [27]. This study was meant to complement a fairly complete understanding of how waterbirds made use of the IBA [27]. Monitoring was conducted and organized by the Mississippi River Twin Cities IBA Landbird Monitoring Program [27,28]. Ten publicly accessible sites within the Mississippi River Twin Cities IBA were monitored by experienced citizen-science birders using 10 to 14 randomly selected point count locations within each site, with 250m distance between sampling points [28]. At each sampling point, all birds seen or heard within 5 minutes were recorded; although data were recorded in 2 distance bands (≤50 m and >50m) [28], data were collapsed into a single total count by species for this analysis. Each site was surveyed 6 times (once weekly) during spring migration from 2007 to 2010 [28].

The IBA point count protocol was designed to create as robust a sampling of landbirds in the target sites as was possible with a select pool of citizen science surveyors. All participants in data collection were recruited from a pool of experienced birders and were required to attend a 2 hour training session which included field practice estimating distance bands and conducting point counts per the required protocol. The training also addressed issues such as double-counting and the value of working in teams with a counter and a data recorder. Sampling design for these point counts was intended to maximize coverage of the sites while ensuring that each could be reasonably sampled in a single morning in order to better facilitate weekly visits during spring migration. Volunteers counted at the same site for the whole season, allowing them to build familiarity with the exact location of each sampling point and distance bands associated with each point.

For this analysis, all monitoring sites along the Mississippi River were merged; whether a bird was found slightly north, slightly south, or in the middle of the Metro area should not affect the potential to encounter some of the buildings being monitored by BirdSafe efforts (Fig 1). Point counts were also merged for each week because monitoring dates varied by volunteer availability and weather conditions.

The percent of the total community was calculated as a measure of relative abundance of each species for each week it was present in the flyway. This number was calculated as the total number of individuals of that species recorded in a given week divided by the total number of individual birds of all species recorded in the same week. Permanent residents were excluded from the all species count to ensure only species with concurrent and overlapping abundances between collision and point count monitoring locations were included in analysis, see *Species included in analysis* section for further discussion. This measurement was then centered around the mean (i.e. rescaled to the mean) and scaled by standard deviation to allow for proper comparison between different continuous variables in the modeling process, hereafter adjusted abundance. Calculating adjusted abundance on a weekly basis allowed for comparison of numbers of collisions while accounting for differences in relative abundance between species without incurring errors in adjusted abundance caused by differences in detection probability across the spring migration season and between years. The potential for other differences in detection probability to affect the analysis was considered with a post-hoc test of the robustness of species-level risk classifications (S1 Appendix, S2 Table).

#### IBA ethics statement

All point counts were conducted on public land and involved passive monitoring via point counts; as such no animals were manipulated in an active way. Such observational only studies do not require oversight from an Institutional Animal Care and Use Committee or other animal ethics management committee. Therefore no Institutional Animal Care and Use Committee protocols were assigned nor any permits required for the IBA point count protocol.

### Species included in analysis

All species recorded in either BirdSafe or IBA monitoring were included in the analysis except for permanent resident species as defined by the Audubon Minnesota’s Bird Species Checklist (Lee A. Pfannmuller compiler). While it means our analysis will not be able to determine the collision risk for permanent residents, the chances of a bias in our analysis for permanent residents were notable, while the chance of a bias for migratory species due to the small distance between our point count locations and our collision locations seemed comparatively minimal.

While the point count monitoring sites and the building collision sites were relatively close to each other (2–36 km), any species that is not at all migratory would not be expected to regularly move between these distances sites in early spring. Thus any permanent residents of the Mississippi River corridor would artificially appear to be superavoiders in the analysis because they did not travel to the areas where the buildings were monitored and permanent residents along the building monitoring routes would appear to be supercolliders because there would be rarely observed in a point count. There are likely individuals of the permanent resident species that live within the monitored collisions areas, but their densities in these areas are unknown compared to the point count monitoring sites. In contrast, given the known movement patterns of migratory birds, it is reasonable to assume that their relative abundances would be similar between the point count locations and the collision monitoring locations as the distance between these sites is well within the expected weekly movement in spring of these birds. Therefore, all permanent resident species were removed from both datasets prior to analysis (Lee A. Pfannmuller compiler). The process removed 381 of 1944 merged records of 15 species, but only removed 32 recorded collisions (S3 Table).

### Merged dataset

Records were aligned using the date of record (shifted to first day of week) and matched for each species. If a species was found in the point count but not in the collision records, the collision value for that species was assumed to be zero for that week. Given evidence that detection probability of dead birds is fairly constant across all species [4], we felt confident that a zero in collision data was a reflection of relatively low or missing collisions by that species.

However, if a species was found in a collision record but not in a point count record for a given week, that data point was dropped from the analysis due to uncertainty about the cause of zero detection. Using point count data as an index of abundance is contested in instances where detection probability might vary ([29–31], but see [32]). In particular, detection probability is likely to vary among species (especially differential visibility and song detection), making comparisons among species problematic [33]. Because of this uncertainty about the cause of zero detection in the point count data (low abundance of the species or low detection probability of the species), we dropped these values from our analysis. This process removed 88 of 1563 records (S4 Table). In contrast, 1297 of the remaining 1475 records showed the presence of birds in the point counts but not in the collision records. This process only eliminated 5 species completely from the analysis (S4 Table), and these are species that are predominantly either nocturnal (such as Eastern Whip-poor-will, *Caprimulgus vociferus*) or challenging to detect during point counts (such as Rust Blackbird, *Euphagus carolinus*). While the elimination of these species means that they could not be assessed for collision risk, it is reasonable to exclude these species for which the point count data is most likely underrepresenting these species and when there are only 5 removed species, leaving 123 species that are more reasonable to analyze. This restriction process will also remove error due to a lack of knowledge about the cause of a zero-value for the relative abundance, but it will lead the final estimates of collision risk to be more conservative; specifically, the shrinkage estimates (estimates of variance) of collision risk will be larger for species with a week’s data point removed from the analysis compared to a species with no weeks’ data points removed from analysis. We determined that a more conservative analysis was preferable to incurring an unknown direction of bias in the analysis.

### Base model of collisions and abundance

Unlike the 2 previous studies [2,4], we used a slightly different approach to the analysis of susceptibility. Previous analysis fixed the slope at 1 on a log-log graph of collisions and abundance and for each species, measured the residual of the species from this idealized line [4]. We still used a regression model between the relative abundances and collisions by species (and by week), but rather than fixing the slope at one, we incorporated into the model random effects for taxonomic groups, and fixed effects for behavioral factors and time of year. In part, this was possible because we had multiple data points per species due to the weekly nature of our dataset across 4 spring migratory seasons. We interpreted the effect of these factors on susceptibility based on how each significant factor changed the intercept, with increased intercepts indicating increased susceptibility to building collisions. Thus the error incurred by interpreting the value of the slope estimated using OLS models that assume error only in the dependent variable will not affect our findings [4,34].

**Fig 2:**
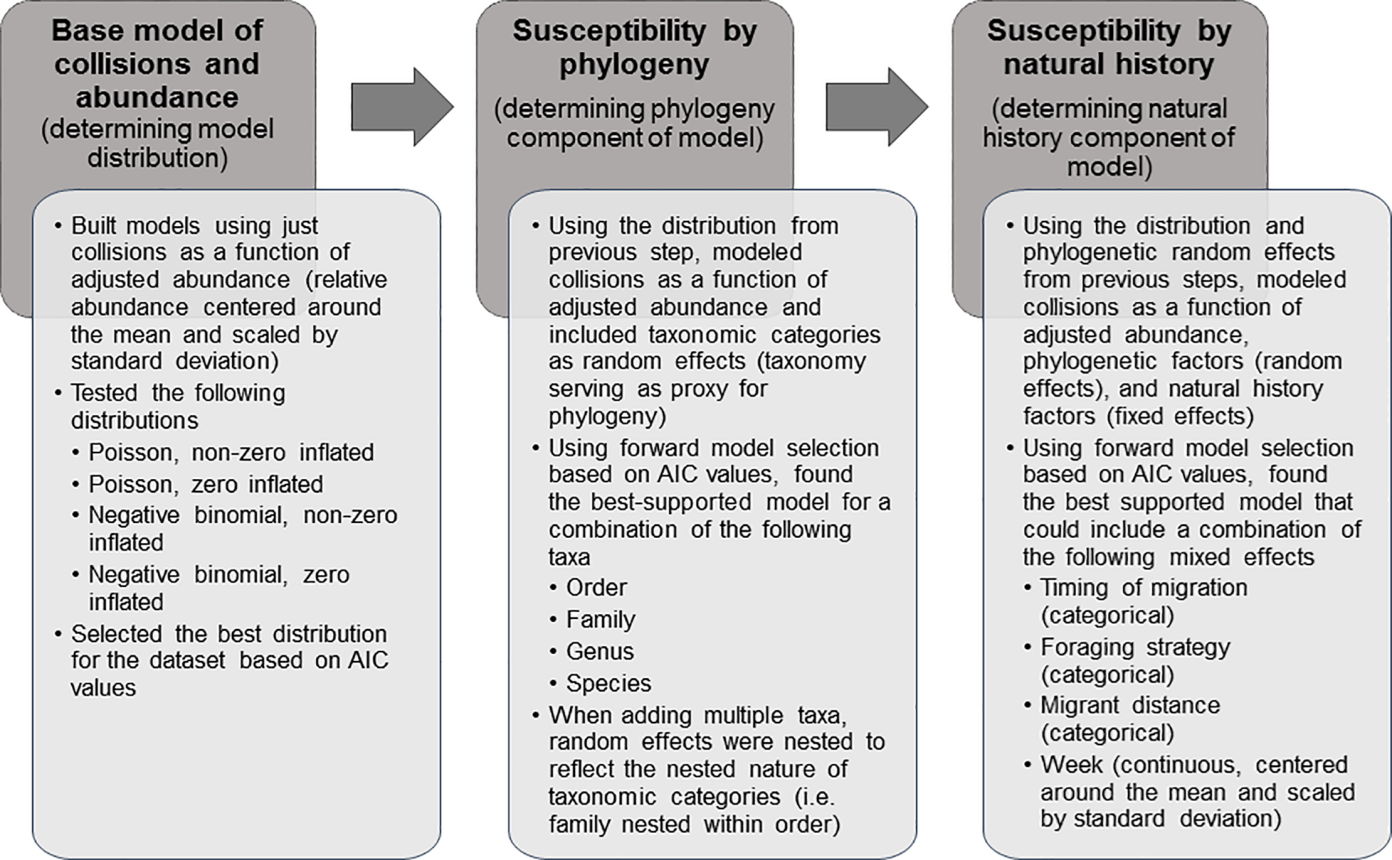
Flowchart diagraming modeling process. Modeling was completed in 3 stages; the first determined the best distribution to use for the dataset, the second determined the most parsimonious combination of phylogenetic taxa, and the third determined which natural history fixed effects were present in the most strongly supported models. All model selection was based on the information-theoretic approach, and used Akaike Information Criterion (AIC) values to determine the best model.

Given the count nature of our collision data, 4 distributions of modeling were tested using the GlmmADMB package in R on the basic regression between collisions and adjusted abundance: Poisson (zero-inflated and non-zero-inflated) and negative binomial (zero-inflated and non-zero inflated) (Fig 2; [35,36]). Model selection was based on the information-theoretic approach, and used Akaike Information Criterion (AIC) values to determine the best model. Throughout all analyses, AIC rather than AIC corrected for sample size, AIC_C_, was used as the sample size, N, divided by the number of parameters in a model, K, was much greater than 40 for all models tested [37]. Of the 4 tested distributions, a negative binomial distribution without a zero inflation had the best fit with the data as determined by AIC values (AIC = 1599.4). Both Poisson and Poisson with zero inflation models were non-competitive (AIC = 2163.8, and AIC = 1723.5, respectively). Although a zero-inflated negative binomial model was apparently competitive (AIC = 1601.4), this model simply contained one additional uninformative parameter [38,39]. Therefore, a negative binomial model from the GlmmADMB package in RStudio 0.98.109 (using R 3.1.2) was used for all subsequent model testing [35,36,40,41].

### Susceptibility by phylogeny

We tested taxonomic categories (order, family, genus, and species) by creating models with each category added as a random effect to the base model to create 4 new models (Fig 2). Taxonomic classifications serve as a decent approximation for phylogenetic relatedness, and we used them as a measure of phylogeny and phylogenetic relationship within our analysis [42,43]. We also tested the phylogenetic relationships by creating models where the species random effect was nested within 1, 2, or 3 successively higher taxonomic levels (order, family, and genus). We then used an information-theoretic approach and compared the AIC values of all of these models to that of the base model to determine if any single phylogenetic category or nested phylogenetic model did a better job of explaining the data than the base, and if multiple models were an improvement, which model had the most support based on AIC values.

### Susceptibility by natural history

Using the best model from the tests of susceptibility by species and phylogeny, we added fixed effect variables that encompassed different behavioral traits that may influence susceptibility to collisions using forward model selection with an information-theoretic approach to determine the most important combination of behavioral traits that may affect collisions (Fig 2). The behavioral traits considered were 1) the time of day a species migrates (hereafter timing), 2) the foraging behavior of a species (hereafter foraging), 3) the distance a species migrates (hereafter migrant), and 4) the week of the year that a collision record occurred (hereafter week). The week variable was centered around the mean value and scaled by standard deviation prior to inclusion in the model. The week variable was only included as a linear variable as there was no indication in the literature that there would be any expected relationship between differential susceptibility and time of the migration season. There was insufficient statistical power to test for differences over time in susceptibility by species, i.e. an interaction effect; with the 4 spring seasons of data, if we segregated data by species and by week, there would only be up to 4 data points per species-week group. Timing was limited to diurnal (including mostly diurnal and diurnal migrants), nocturnal (including mostly nocturnal and nocturnal migrants), or other migrants (including birds that migrate both day and night, birds classified as non-migratory, and birds with unknown migratory timing) to retain statistical power. Similarly, migrant was limited to short distance, long distance, or other migratory strategy to retain statistical power. While permanent resident species had previously been removed from the analysis, some species that are classified as other remained in the dataset. These were species for which there is some debate about their annual movement patterns; for example White-Breasted Nuthatches (*Sitta carolinensis*) were originally classified as non-migratory and then as other, but due to a tendency to join mixed species winter foraging flocks and intermittent winter dispersals they were not classified as a permanent resident species to exclude from analysis [44]. Classifications were provided by Arnold (S4 Table; [4]). For those species that were missing from the previously described dataset (Alder Flycatcher (*Empidonax alnorum*), Double-crested Cormorant (*Phalacrocorax auritus*), Harris's Sparrow (*Zonotrichia querula*), Killdeer (*Charadrius vociferous*), Mallard (*Anas platyrhynchos*), Sora (*Porzana carolina*), Virginia Rail (*Rallus limicola*), and Wood Duck (*Aix sponsa*)), the natural history factors were determined based on the species records within Birds of North America Online (S5 Table; [45]).

No interactions between effects were tested in our analysis; for many of these interactions there is limited, if any, research to support an ecological explanation for an interaction term. In the case of a week-species interaction, there is reason to suppose that the time within the migration window could affect the susceptibility of each species differently, particularly as different species have different timings of migration and some species have separation of migration between age classes or sexes. However, as the analysis contained data from 4 years, there would be at most 4 data points per week-species category; such a limited sample size was deemed too small to merit an analysis at this time.

### Classification of supercolliders and superavoiders

Once the best model (or models) was found, we assessed which, if any, species, genera, families, or orders of birds seem to collide or avoid collisions much more than would be expected based on their abundances. As coined by Arnold and Zink [4], supercollider and superavoider are used to identify those species that are most and least likely to collide with buildings in proportion to their abundance. As these terms have been used in subsequent assessments of species susceptibility [2], we will also use these terms for consistency of language within the field of study. For our analysis, we will classify supercolliders and superavoiders based on the random effect coefficients and the shrinkage estimates (a measure of variance of each random effect coefficient estimate produced by the model) in the top fitting model. All species for which the random effect estimate minus the shrinkage estimate were greater than zero were classified as supercolliders (i.e. the random effect estimated range was completely greater than zero). All species for which the random effect estimate plus the shrinkage estimate were less than zero were classified as superavoiders (i.e. the random effect estimate was completely less than zero). In other words, only species with a random effect estimate ± shrinkage estimate that did not include zero were classified as superavoiders or supercolliders.

Following determination of the best supported model, a post-hoc test was done on the robustness of random effect estimates of species to errors in detection probability and determined that the random effect estimates were quite robust to unaccounted variability in abundance due to variation in detection probability (S1 Appendix, S2 Table).

### Comparisons with previous species risk assessments

To compare the risk assessment of species from this analysis with those previously published, we analyzed our results and previous results in 3 ways. For the first two tests, we assessed the correlation between the net random effect of each species (random effect estimates of species, genus, and family summed for each species for the best fitting model) and the risk valuation by species from Arnold and Zink [4] and from Loss et al. [2]. When testing with Arnold and Zink’s vulnerability assessment, there were 114 species in common with our analysis [4]. For Loss et al., we used the collision risk values based on all buildings–although all of the buildings in our study were located in urban areas, the building structures were not limited to high-rises, thus a comparison with the all building analysis seemed more logical [2]. There were 105 species in common with our analysis [2]. Similar analyses using only the random effect estimates of species from our best model did not produce substantively different results, and since neither Arnold and Zink [4] nor Loss et al. [2] separated the phylogenetic component from their species risk assessment, a comparison with our net random effect (including genus and family random effects with each species effect) seemed a more parallel comparison.

As a third assessment of our analyses, we condensed our abundance and collision data and analyzed it mimicking the analysis done by Arnold and Zink [4]. This analysis allowed us to verify that any differences observed by the above comparisons were due to substantive differences of spatially localized abundance data rather than a product of the variation in our analytical techniques. All percent abundances for each species were averaged and all collision records for each species were summed. These values were log transformed (using log_10_(X+1) to allow for zeros), and then the residual from a theoretical linear slope of 1.0 was found for every species [4]. This residual was used to represent the relative risk of collision by species and was compared to the collision risk estimates based on the random effect estimates from our best-fit model with a simple correlation analysis.

Calculations of data to mimic Arnold and Zink’s analysis were done in Microsoft Excel (Excel 2013). All correlation analyses were conducted in in RStudio 0.98.109, using R 3.1.2, as Pearson-product moment correlations [40,41].

## Results

### Susceptibility by phylogeny

Of the 4 levels of taxonomic grouping that were tested, species nested within genus within family was the best fit (Table 1). Modeled data included 123 species from 77 genera, 32 families, and 13 orders. Mean sample size per category was 12.0 for species, 19.1 for genera, 46.1 for families, and 113.5 for orders.

**Table 1:** Table of the 4 taxonomic models tested with ΔAIC values as well as a null model.

Random effect added	ΔAIC^a^	Log-Likelihood	K
Family/Genus/Species	0	-644.83	6
Order/Family/Genus/Species	2.0	-644.83	7
Genus/Species	2.1	-646.90	5
Species	4.4	-649.07	4
Null model	297.7	-796.69	3

Null Model is the negative binomial regression of Collisions as a function of Abundance. All other models add a random effect to the null model with / used to indicate nested random effects. The top model based on ΔAIC is the model with Family/Genus/Species as the random effect. K represents the number of parameters in each model.

^a^ΔAIC calculated based on minimum AIC value amongst all models. Minimum AIC value was 1301.7 for the model with Family/Genus/Species as a random effect.

### Susceptibility by natural history

The top models examining natural history characteristics all included abundance, timing of migration (e.g., nocturnal vs. diurnal), and family/genus/species as a random effect (Table 2). The highest ranked model by AIC also included week of migration, however the simpler model (without week) is highly competitive with this model (ΔAIC -0.6) and avoids the risk of over-fitting the model [38]. Additionally, the coefficient estimate of the week parameter was small (-0.151±0.096) and non-significant compared to the timing behavior coefficient estimates. While foraging behavior and migratory distance were in the top models, they both appear to be “uninformative parameters” such that a simpler model has a better fit as assessed by AIC (Table 2; [38]).

**Table 2:** Table of the forward model selection results with the base phylogeny model and the top models from the model selection process, based on minimum ΔAIC.

Model	ΔAIC^a^	Log-Likelihood	K
Abundance+Timing+Week+(1|Family/Genus/Species)	0	-633.77	9
Abundance+Timing+(1|Family/Genus/Species)	0.6	-635.03	8
Abundance+Timing+Week+Migrant+(1|Family/Genus/Species)	3.0	-633.26	11
Abundance+Timing+Week+Forage+(1|Family/Genus/Species)	3.2	-629.34	15
Abundance+Timing+Migrant+(1|Family/Genus/Species)	3.3	-634.42	10
Abundance+Timing+Forage+(1|Family/Genus/Species)	3.4	-630.45	14
Abundance+(1|Family/Genus/Species)	16.2	-644.83	6

Model descriptions show the variables used in each model to predict Collisions using a negative binomial regression function. Of the 9 models calculated in the analysis, only the models which had ΔAIC<9.0, are shown, in addition to the starting model of Abundance + (1|Family/Genus/Species). The model with the lowest AIC was Abundance+Timing+Week+(1|Family/Genus/Species). The model Abundance+Timing+(1|Family/Genus/Species) was highly competitive. K represents the number of parameters in each model.

^a^ΔAIC calculated based on minimum AIC value amongst all models. Minimum AIC value was 1285.5 for the model Abundance+Timing+Week+(1|Family/Genus/Species).

Based on the best model, *Collisions* ~ *Adjusted Abundance* + *Timing* + (1|*Family/Genus/Species*), increased abundance correlates with increased collision risk (coefficient = 0.435±0.094), nocturnal migrants are more likely to collide than diurnal migrants (coefficient = 2.651±0.538), and other migrants are more likely to collide than diurnal migrants (coefficient = 1.705±0.626). Based on this same model, and assuming a negative binomial distribution of the response variable, the mean collisions for a given species(*i*) that is a nocturnal migrant can be expressed as:

$${Collisions}_{i}=e^{-1.993+0.435\times {Adjusted\;Abundance}_{i}+{\hat{\alpha }}_{Family}[{Family}_{i}]+{\hat{\alpha }}_{Genus}[{Genus}_{i}]+{\hat{\alpha }}_{Species}[{Species}_{i}]}.$$

### Classification of superavoiders and supercolliders

**Fig 3:**
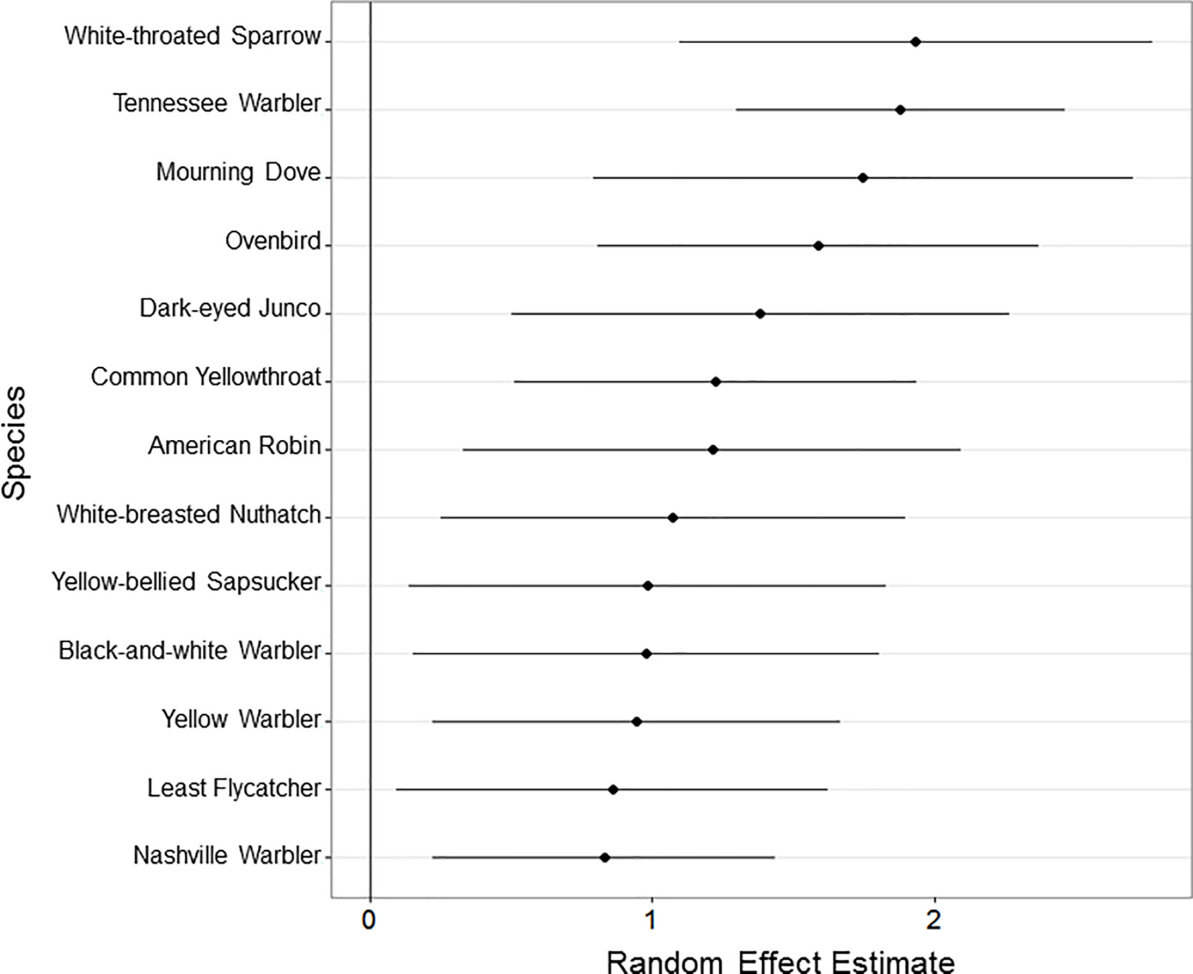
Caterpillar plot of the 13 supercollider species random effects estimates ± shrinkage estimates within the top model: Abundance+Timing+(1|Family/Genus/Species). Random effect estimates indicate the estimated level of risk for each species after accounting for the effects of Abundance and Timing as approximated by the model. Shrinkage estimates are a measure of variance for the random effect estimates. Species were classified as supercolliders when *random effect estimates – shrinkage estimates* > 0, indicating high confidence in a non-zero estimate of risk of collisions greater than expected by Abundance and Timing alone. Within the supercolliding species, there is a clear gradient from highest risk, White-throated Sparrow (*Zonotrichia albicollis*) and Tennessee Warbler (*Leiothlypis peregrine*), to lower risk, Nashville Warbler (*Leiothlypis ruficapilla*) and Least Flycatcher (*Empidonax minimus*).

Within the highest ranked model, no family of birds has a random effect estimate that significantly deviates from zero, but one family, Parulidae or New World Warblers, appears to have a much larger level of variation in collision risk based on shrinkage estimates than all other families (S1 Fig). The coefficient and shrinkage estimates of random effects of species and genera indicate that 13 species (Figs 3 and S2) and 7 genera, *Seiurus*, *Zenaida*, *Zonotrichia*, *Junco*, *Turdus*, *Geothlypis*, and *Vermivora* (S3 Fig), are more likely to hit buildings than predicted, while 1 species, Field Sparrow (*Spizella pusilla*) (S2 Fig), and 1 genus, *Vireo*, (S3 Fig) are less likely to hit buildings. It is important to note that 3 of these genera (*Zenaida* or Zenaida doves, *Junco*, and *Turdus* or thrushes) are single species genera with the species included in this analysis. Additionally, *Seiurus* or ovenbirds and *Zonotrichia* or American sparrows are 2-species genera with the species included in this analysis.

### Comparisons with previous species risk assessments

**Fig 4:**
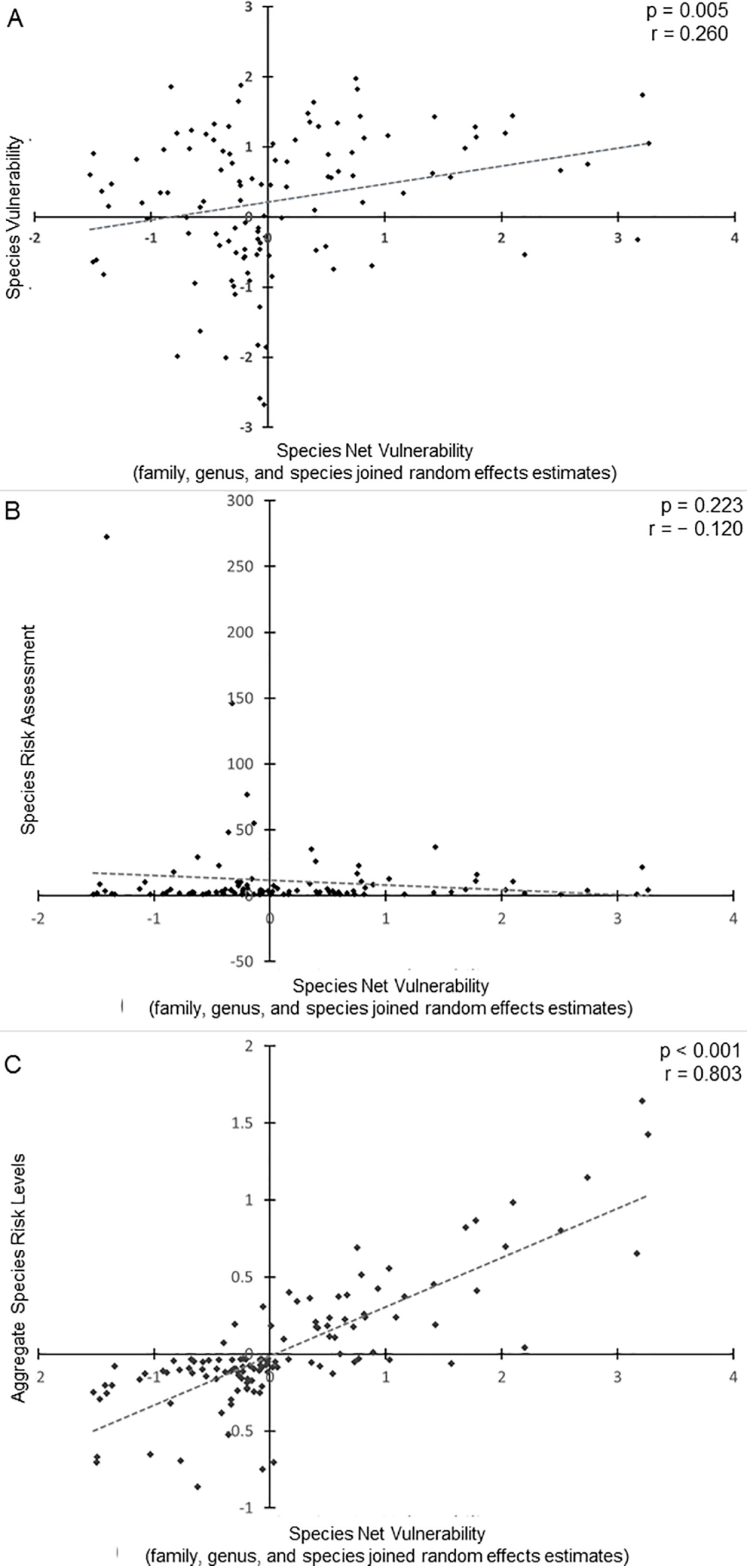
Scatterplots of correlations of previous species risk assessments with the species net vulnerabilities from our analysis. P-value, Pearson product-moment correlation (r), and linear trendline shown for each graph. Relative abundance data for our analysis came from point counts conducted within 36 km of the location of collisions. Collision data came from daily monitoring of buildings along two highly urban routes within Minneapolis and St. Paul, MN, USA. (**A**) Species vulnerabilities calculated by Arnold and Zink [4] are weakly correlated with our species net vulnerabilities. Arnold and Zink used Partners in Flight estimates to determine relative abundances of species and used collision data from select buildings as well as communication towers for their analysis [4]. (**B**) Species risk assessment calculated by Loss et al. [2] was not correlated with our species net vulnerabilities. Loss et al. also used Breeding Bird Survey data to assess relative abundances of species but collision data came from building collisions in residential and urban areas throughout North America [2]. (**C**) Aggregate species risk levels (calculated by using our data and mimicking the Arnold and Zink analysis [4]) are strongly correlated with our species net vulnerabilities.

There was a significant but weak correlation between our net random effects per species and the species vulnerability estimates calculated by Arnold and Zink (*p* = 0.005, *r* = 0.260), (Fig 4A; [4]). There was no significant correlation between our net random effects per species and the species risk assessment values found by Loss et al. (*p* = 0.223, *r* = −0.120), (Fig 4B; [2]). There was a strong significant correlation between the net random effects per species and the species risk calculation modeled after Arnold and Zink (*p*<0.001, *r* = 0.803), (Fig 4C; [4]). Thus, while our risk assessments are very similar to a coarser analysis of our own data and somewhat similar to the original Arnold and Zink [4] analysis, they are quite different from the Loss et al. [2] assessment of species vulnerabilities. The net random effect estimates produced by summing the random effect estimates for species, genus, and family can also be converted into a measure of effect size per species if all other model factors were held constant (S6 Table).

## Discussion

Recent research [2,4] contradicts early papers [9] with regards to the differential susceptibility to bird-building collisions. Our analysis of species susceptibility to collisions using 4 years of collision data and spatially and temporally coinciding abundance data revealed that after accounting for local abundance, building collisions are selective by several taxonomic categories, particularly genus and species, and that the timing of migration may also affect collision susceptibility.

As expected, our top model shows that an increase in abundance is associated with an increase in collisions–if there are more individuals of a species around, more birds of that species will hit buildings. Specifically, all other variables being constant, a one unit increase in the adjusted abundance (the relative abundance values that were centered around the mean and scaled by standard deviation prior to analysis) translates to a 0.435 increase in the logarithm of expected collisions index.

In addition to abundance, our study identified one behavioral trait that is associated with collision risk. Nighttime and other migrants are more likely to collide with buildings than diurnal migrants. Specifically, the coefficient estimate for nighttime migrants was 2.651 ± 0.538 (coefficient estimate ± standard error, p<0.001) and the coefficient estimate for other migrants was 1.705 ± 0.626 (coefficient estimate ± standard error, p = 0.007) compared to daytime migrants. This is consistent with the findings of Arnold and Zink in terms of time of day of migration and collision risk where nighttime migrants were 10.9 times more likely to hit buildings than daytime migrants [4]. These findings suggest that there is a behavioral or physiological difference in daytime migrants that affords them a greater ability to avoid building collisions. Nighttime migrants experience sleep deprivation, and while major declines in cognitive function have not been found under laboratory conditions [46], these birds may be less adept at interpreting the visual cues indicative of building glass. This sleep deprivation has been shown to alter the diurnal foraging behavior to include micro-naps in Swainson’s Thrushes (*Catharus ustulatus*) [47]; perhaps sleep deprivation causes other behavioral changes in nighttime migrants that makes these birds more likely to hit buildings. It is important to note that while nighttime migrants appear more susceptible to collisions, we have no evidence that these collisions are occurring at night. In fact, previous research shows that collision frequency peaks in early morning when considering all species in aggregate [9,11,48,49] and this is also consistent with volunteer observations along BirdSafe routes in Minneapolis and Saint Paul [J. Eckles personal observation]. If nighttime migrant collisions also peak in early morning, this would support the hypothesis that nighttime migrants are hitting buildings when they are physically and mentally depleted after a night of migration and are searching for food and/or roosting sites rather than hitting buildings during migration flights. Additionally, as nighttime migrants are selecting stopover habitat under low light conditions, they are less able to visually assess habitat and thus avoid areas with high densities of buildings compared to daytime migrants. Furthermore, nighttime migrants may be attracted to urban areas because of light pollution by buildings and are therefore selectively choosing stopover habitat in closer proximity to buildings than diurnal migrants.

In our analysis, no other behavioral traits were found to be significantly associated with collision risk. However, Arnold and Zink [4] found migration distance to influence collision risk. Intuitively, longer-distance migrants would encounter more buildings because they cover a greater distance during migration and therefore have a cumulative collision risk greater than short-distance migrants. However, in the Arnold and Zink analysis, collision risk is based on the risk of collision per building or per city, which would not include this cumulative effect [4]. Thus the Arnold and Zink analysis suggests that there is a behavioral or physiological difference in long-distance migrants that makes them more likely to collide with buildings [4]. Our analysis was unable to replicate this finding, suggesting that the effect of migration distance is small relative to the other factors affecting collisions risk. Alternatively, by incorporating taxonomy in our analysis prior to natural history characteristics, we were able to capture the variation Arnold and Zink attributed to migration distance within taxonomic classifications. It may be that migration distance does affect collision risk and that migration distance corresponds sufficiently closely to taxonomic classifications that in our analysis the effect of migration distance was masked by taxonomy. Or it may be that both migration distance and taxonomy correlate with some, as yet untested, factor that is a more genuine driver of collision risk variation among species. Nonetheless, the cumulative effect of encountering multiple buildings during migration and the substantial risk that may accrue for long-distance migrants should not be ignored.

Our modeling also shows that including species as a model variable significantly improves model performance, based on AIC scores. While over 80% of species considered in the model are neither more nor less likely to collide with windows than expected after accounting for timing of migration and local abundance, this estimate is a conservative one that will overlook particularly rare species which will have very large shrinkage estimates that exclude them from being considered supercolliders or superavoiders. For example, the Golden-winged Warbler (*Vermivora chrysoptera*) is a Near Threatened species on the IUCN red list [50], and had a risk level of -0.499 ± 0.924 (random effect estimate ± shrinkage estimate); the large shrinkage estimate for the species–in part due to the small sample size (n = 7)–made it impossible to classify this species as a superavoider, despite a trend towards avoidance (S2 Fig). While acknowledging the conservativeness of our classification of superavoiders and supercolliders, the relatively few obvious supercolliders and superavoiders may explain why early work on susceptibility to collisions, which relied on more limited datasets, found no differential susceptibility to collisions by species [9]. Were our analysis to be replicated in other locations across North America, particularly in locations in the Western part of the continent, where avian communities are different from the Upper Midwest, we would expect some differences in the particular species that are classified as superavoiders or supercolliders.

Our findings also corroborate the more recent work on susceptibility by species that relied on national abundance metrics, demonstrating that some species are much more susceptible to collisions than can be explained by their abundance and others are much less susceptible to collisions [2,4]. In contrast to those other studies, our analysis is also able to show a clear taxonomic connection to collision risk at higher phylogenetic levels than species; both family and genus classifications were part of our top model. Previous work was only able to demonstrate increased collision risk for 1 family and decreased collision risk for a second [4]. Within our analysis, Parulidae are more variable in their collision risk than any other family. The variability we observed is likely explained by the diversity of collision risks for the Parulidae genera in the analysis: 3 genera were classified as supercolliders (*Seiurus* or Ovenbirds, *Geothlypis* or Yellowthroats, and *Vermivora*); 2 more genera had positive random effect estimates that did not qualify as supercolliders (*Mniotilta* and *Setophaga*); and the remaining 4 genera had negative random effect estimates though they did not qualify as superavoiders (*Dendroica*, *Parula*, *Protonotaria*, and *Wilsonia*). In addition to 3 warbler genera, 4 other genera of birds are supercolliders: *Zenaida*, *Zonotrichia*, *Junco*, and *Turdus*. In contrast, only one genus, *Vireo*, is a superavoider. The extent of these higher order taxonomic links to collision risk suggests that there may be evolutionary explanations for why some birds hit buildings more than others. There may be some behavioral or morphological shift(s) in the evolutionary history of *Seiurus*, *Geothlypis*, and *Vermivora* that not only distinguishes them from other Parulidae, but also makes them more susceptible to bird-building collisions. However, such conclusions should be tempered by the often small number of species within many of these genera–in some cases only 1 or 2 species were included in a genus as part of this analysis–which may have resulted in species-level differences in collision risk being attributed to the genus-level as part of the modeling process.

There was a high correlation between the risk assessment based on random effects and that based on the Arnold and Zink [4] style of assessment (*r* = 0.803), (Fig 4C), suggesting that the dataset determines the risk assessment outcome more than the analysis choice. While there was a significant correlation with Arnold and Zink [4] and a non-significant correlation with Loss et al. [2], both correlations were weak and overall reveal that the risk assessments found with our data are different from those found previously (*r* = 0.260,– 0.120, Fig 4A and 4B, respectively). While no formal analytic comparison was possible with the other localized study of species susceptibility, and informal comparison showed substantial differences; for example, Wittig et al. [26] identified Vireonidae as colliding more than expected based on abundance while we classified *Vireo* as a superavoiding genus. There are 3 possible reasons for these differences with national-scale analyses. The first is seasonality; our analysis only used spring migration while both Arnold and Zink [4] and Loss et al. [2] used data from spring and fall migration periods. The age distribution of spring migrants is decidedly different from fall migrations and could lead to behavioral shifts for some species that would change their risk assessments, but it seems unlikely that this could account for the substantial shifts between risk assessments in both directions that were seen in the comparison between Loss et al. [2] and our assessment (Fig 4B). The second possible explanation is the composition of the buildings used in the analyses. Arnold and Zink used solely urban data from 3 cities where monitoring focused on high-collision buildings [4]. Loss et al used data from buildings across the urban to rural gradient [2]. Our analysis used urban buildings with a range of collision rates. If species susceptibility varies based on building or landscape features–and Loss et al. provide some evidence that it does [2]–then the buildings used for determining collision numbers could be affecting the species risk assessment. Further research is needed to comprehensively test if and how differential species susceptibility to collisions changes with building or landscape changes. The third and most likely explanation for the differences among the 3 analyses is that the abundance data used in the 3 studies were substantially different. Both previous studies relied on large regional measures of abundance using BBS [2,4]. Our study made use of locally collected data that were also temporally specific within season. While a comparison between 2 analytical techniques (one of which does not include temporal information) with our data suggests that temporal specificity within season has only marginal effects on the resulting risk assessment (r = 0.803, Fig 4C), the substantial differences between our assessment results and those of previous research suggests that the local specificity of abundance data plays an important role in the results of the risk assessment.

In a perfect world, when testing the differential susceptibility of species to building collisions, a researcher wants a snapshot of the total number of birds of each species present in the area the night before and the day of the records of collision. However, the tools to capture such a snapshot do not yet exist, especially within the budget considerations of bird-building collision research. While imperfect, point count data along the major migration and habitat corridor in the area of collisions measured within the same week as those collisions are a reasonably good means of measuring local abundances and testing which species are most at risk and least at risk of colliding with windows, as long as analyses acknowledge that some species cannot be analyzed in this fashion if point-count data will be poor at detecting these species (such as nocturnal or especially secretive species). Compared to prior analyses, our analysis was able to match abundance and collisions at weekly and regional scales rather than aggregating collisions across entire years and matching migratory season collisions with abundance records for breeding seasons in areas northward of the location of the collisions [2,4–6]. While we have not yet achieved the idealized study, we have come much closer than any previous analysis.

Both of our datasets were collected by Citizen Scientists, which are notorious for being of poorer quality than data collected by paid monitors/professional scientists [51,52]. This poor quality reputation is not always justified, and that is particularly true of both datasets used in this study [51]; both Citizen Science monitoring programs required relatively intensive training prior to collection of data by volunteers and data entry was managed by the professional organizers of the projects (see Methods). Additionally, in the case of Project BirdSafe (collision monitoring), all species identifications were verified at the end of each season by multiple avian experts prior to data entry.

The differences from previous studies and improved specificity of our analysis, both temporally and spatially, has implications for mitigation of building collisions because our analysis shows that the majority of species do not have variable susceptibility beyond that explained by abundance and timing (diurnal vs nocturnal) of migration and that the best expenditure of efforts for those species is to find ways of modifying or designing buildings that make them more visible and/or less deadly to all species. However, it is also important to identify those species that are most susceptible to building collisions; for these species, generalized mitigation efforts may be insufficient to prevent building collisions. Research that identifies the behavioral or physiological characteristics that affect susceptibility can make it possible to enact targeted modifications of buildings that prevent collisions by these supercolliders. Given the limited research on the physiological and behavioral reasons behind building collisions, research focused on the species and genera identified in this analysis as supercolliders and superavoiders could be helpful in improving our understanding of why birds fly into windows. For example, within Parulidae, Ovenbirds (*Seiurus*) and Yellowthroats (*Geothlypis*) are highly susceptible to collisions while *Protonotaria* and *Wilsonia* are much less susceptible and possibly even avoid building collisions to some extent. Similarly, White-throated Sparrows (*Zonotrichia albicollis*) and Field Sparrows (*S*. *pusilla*) are at opposite ends of the collision risk spectrum despite their relative phylogenetic proximity. Research that focuses on the physiological and behavioral differences between these clades could be especially fruitful to determining what makes some birds more likely to hit buildings than other birds.

## Conclusion

In summary, it is possible to use local point count data to determine relative abundances of species and analyze susceptibility of window collisions in terms of phylogenetic and behavioral characteristics. Our findings from such an analysis confirm that for many species, abundance and timing of migration are the predominant determining factors for collision risk, but that for 20% of species, the species, genus, and family of a bird may affect the collision risk. For these birds, further research is needed to determine what the physiological or behavioral factors are that lead to building collisions and if any of these factors also relate to the phylogenetic trends of collision risk. Potentially, these are species and taxa that need to be the focus of conservation efforts both for mitigating collision mortality and for supporting reproduction and survival throughout the lifecycle of these species to minimize the damage that collisions cause to these populations. Furthermore, understanding these trends in susceptibility can inform the direction for future research and improve understanding of the physiological and behavioral causes of bird-building collisions, thus improving the efficacy of efforts to prevent building collisions–one of the greatest sources of direct avian mortality.

## Supporting information

S1 Fig Caterpillar plot of the family random effects estimates ± shrinkage estimates within the top model: Abundance+Timing+(1|Family/Genus/Species). With the exception of Parulidae, all families have similar random effect estimates. Parulidae has a greater shrinkage estimate than all other groups, 5.6 × 10^−4^, indicating greater variability in the risk of collisions among Parulidae species than among species of any other family.
(TIF)

S2 Fig Caterpillar plot of the species random effects estimates ± shrinkage estimates within the top model: Abundance+Timing+(1|Family/Genus/Species). Thirteen species have random effects estimates ± shrinkage estimates completely greater than zero, indicating supercolliding species and are shown in green. Only one species (Field Sparrow) has a random effects estimate ± shrinkage estimate completely less than zero, indicating a superavoiding species and is shown in purple.
(TIF)

S3 Fig Caterpillar plot of the genus random effects estimates ± shrinkage estimates within the top model: Abundance+Timing+(1|Family/Genus/Species). Only *Vireo* has a random effect estimate ± shrinkage estimate that is completely less than zero, indicating a superavoiding genus and is shown in purple. There are 7 genera that have random effect estimates ± shrinkage estimate that are completely greater than zero, indicating supercolliding genera: *Seiurus*, *Zenaida*, *Zonotrichia*, *Junco*, *Turdus*, *Geothlypis*, and *Vermivora*, and are shown in green. Note that 3 of these genera (*Zenaida* or Zenaida doves, *Junco*, and *Turdus* or thrushes) are single species genera with the species included in this analysis. Additionally, *Seiurus* or ovenbirds and *Zonotrichia* or American sparrows are 2-species genera with the species included in this analysis.
(TIF)

S1 Table Table of collision records of all birds collected by Project BirdSafe during April and May of 2007 through 2010. Every bird collected by Project BirdSafe citizen scientists was recorded and the species identification of all collected carcasses was verified by an expert team prior to data entry. Only birds collected in April and May of the spring monitoring season could be considered for our analysis because these were the only weeks with concurrent abundance data from the IBA monitoring effort. Bird collision records were summed by species and week prior to analysis.
(XLSX)

S2 Table Post-hoc test of robustness to detection probability errors. Random effect estimates (REE) and shrinkage estimates (SE) for each species along with coefficient of abundance from top supported model (0.4349) and the correction factors used to center and scale the relative abundance values (adjusted abundance = 30.697*relative abundance– 0.6451) were used to calculate the change in abundance estimate necessary to result in a change in the classification of a species as supercollider, superavoider, or neither (ΔAbd). The classification of a species would change if REE ± SE for a supercollider or superavoider species was to shift to include 0 and for a species that was neither a supercollider nor superavoider, the classification would change if REE ± SE shifted to no longer include 0. ΔAbd was combined with the average relative abundance for each species to find the percent change in abundance necessary to change the classification of each species. Species are listed in order of increasing percent change. Species with common names in bold are classified as supercolliders or superavoiders.
(DOCX)

S3 Table Table of collisions of all permanent resident species removed from dataset prior to analysis. Numbers represent the total number of individual birds recorded as colliding with a building during springs 2007 through 2010. Those species with no collisions are species that were observed in point counts but had no records of collisions during the study period.
(DOCX)

S4 Table Table of data points removed from analysis for having zero point-count detections in a week but a non-zero number of collisions in the same week. Data removed from analysis due to uncertainty about the cause of the zero point-count detection. Table sorted by species common name and then by the first date of the week for each data point. Number of Collisions indicates the number of collisions by that species that were recorded by Project BirdSafe monitors during the week that started with the date for that row. 88 data points were removed with this restriction, representing 39 different species. Only 5 species were completely eliminated from the analysis with this restriction: American Woodcock (*Scolopax minor*), Eastern Whip-poor-will (*Caprimulgus vociferus*), Fox Sparrow (*Passerella iliaca*), Pied-billed Grebe (*Podilymbus podiceps*), and Rusty Blackbird (*Euphagus carolinus*).
(DOCX)

S5 Table Table of taxonomic classifications, natural history traits, collision, and relative abundance data used in analysis as well as and random effect estimates from top supported model for all species included in analysis. Migrant represents the distance a species migrates: long distance (LD), short distance (SD), or other migratory pattern (Other). Timing represents the time of day a species migrates: diurnal or mostly diurnal (Diurnal), nocturnal or mostly nocturnal (Noct), or other migrants (Other). Foraging represents the foraging behavior of a species: aerial foragers (Aerial), bark gleaners (Bark), foliage gleaners (Foliage), ground foragers (Ground), hawking foragers (Hawks), hover gleaners (HoverGlean), and other foraging strategies (Other). Natural history traits based on information from Arnold (Arnold and Zink 2011) except for the following species, which were completed using the species records from Birds of North America Online: Alder Flycatcher, Double-crested Cormorant, Harris's Sparrow, Killdeer, Mallard, Sora, Virginia Rail, and Wood Duck (Rodewald 2015). Number of collisions represents total number of collisions by species used in analysis, while species specific sample size represents the number of data points included in the model for that species. Average relative abundance represents the average of relative abundance values used across all data points for each species. Random Effect Estimates represent the estimated random effect size for each species, genus, and family. Shrinkage Estimates are a measure of variance of random effect estimates, for each species, genus, and family.
(XLSX)

S6 TableTable of the calculated Incidence-Rate Ratios (IRR) for all species.For each species, the 3 random effects estimates for the Family, Genus, and Species were summed and the sum was then used as the exponent of *e* to calculate the Net IRR. In other words, for each species *i*, ${Net\;IRR}_{i}=e^{{\hat{\alpha }}_{Family}[{Family}_{i}]+{\hat{\alpha }}_{Genus}[{Genus}_{i}]+{\hat{\alpha }}_{Species}[{Species}_{i}]}$. IRR can be interpreted as the number of times it is more likely for that species to collide with a building than the model average. As a specific example, a White-throated Sparrow (*Z*. *albicollis*) is 26.12 times more likely to hit a building than the average nocturnal migrant with the same relative abundance during the same week of spring migration.(DOCX)

S7 Table Table of match-up of monitoring between Project BirdSafe and Mississippi River Twin Cities Important Bird Area Landbird Monitoring Project for spring 2009. Number of routes (for Project BirdSafe) and sites (for IBA) monitored per day during the overlapping spring 2009 season.
(XLSX)

S1 Appendix Post hoc assessment of random effect estimate robustness to detection probability variability. (DOCX)
