# Supplementary figures and images for: Bird-building collision risk: An assessment of the collision risk of birds with buildings by phylogeny and behavior using two citizen-science datasets
Source: PLoS One. 2018 Aug 9;13(8):e0201558. doi: 10.1371/journal.pone.0201558 (PMC6084936; doi:10.1371/journal.pone.0201558)

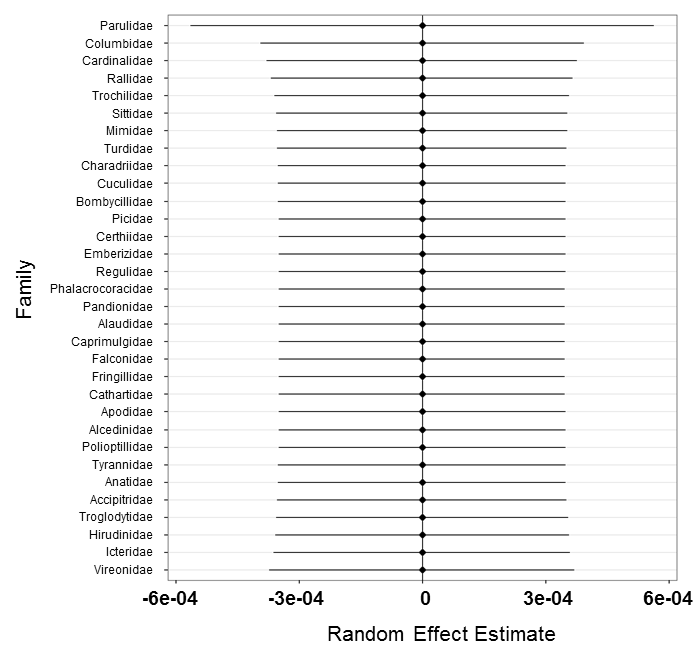

Supplement: S1 Fig — With the exception of Parulidae, all families have similar random effect estimates. Parulidae has a greater shrinkage estimate than all other groups, 5.6 × 10^−4^, indicating greater variability in the risk of collisions among Parulidae species than among species of any other family. (TIF) [file pone.0201558.s001.tif]

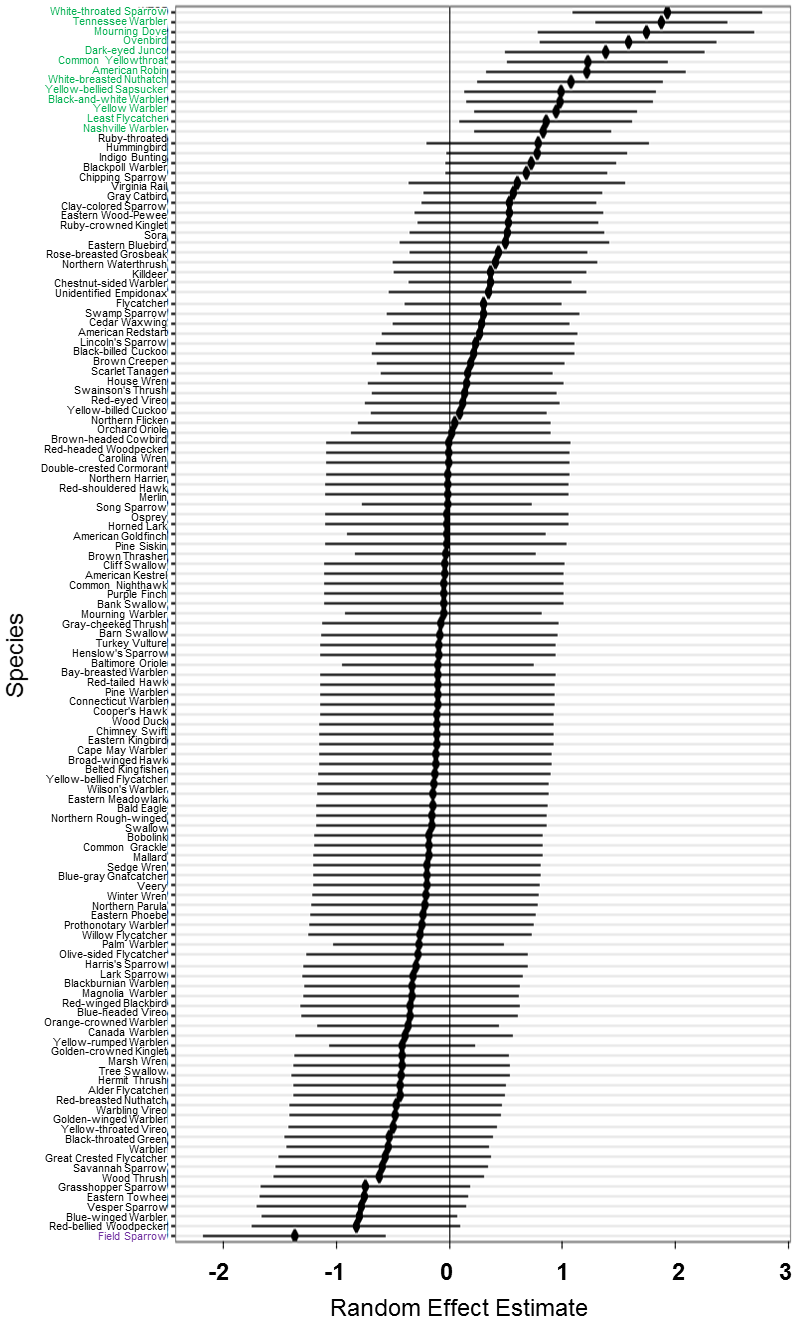

Supplement: S2 Fig — Thirteen species have random effects estimates ± shrinkage estimates completely greater than zero, indicating supercolliding species and are shown in green. Only one species (Field Sparrow) has a random effects estimate ± shrinkage estimate completely less than zero, indicating a superavoiding species and is shown in purple. (TIF) [file pone.0201558.s002.tif]

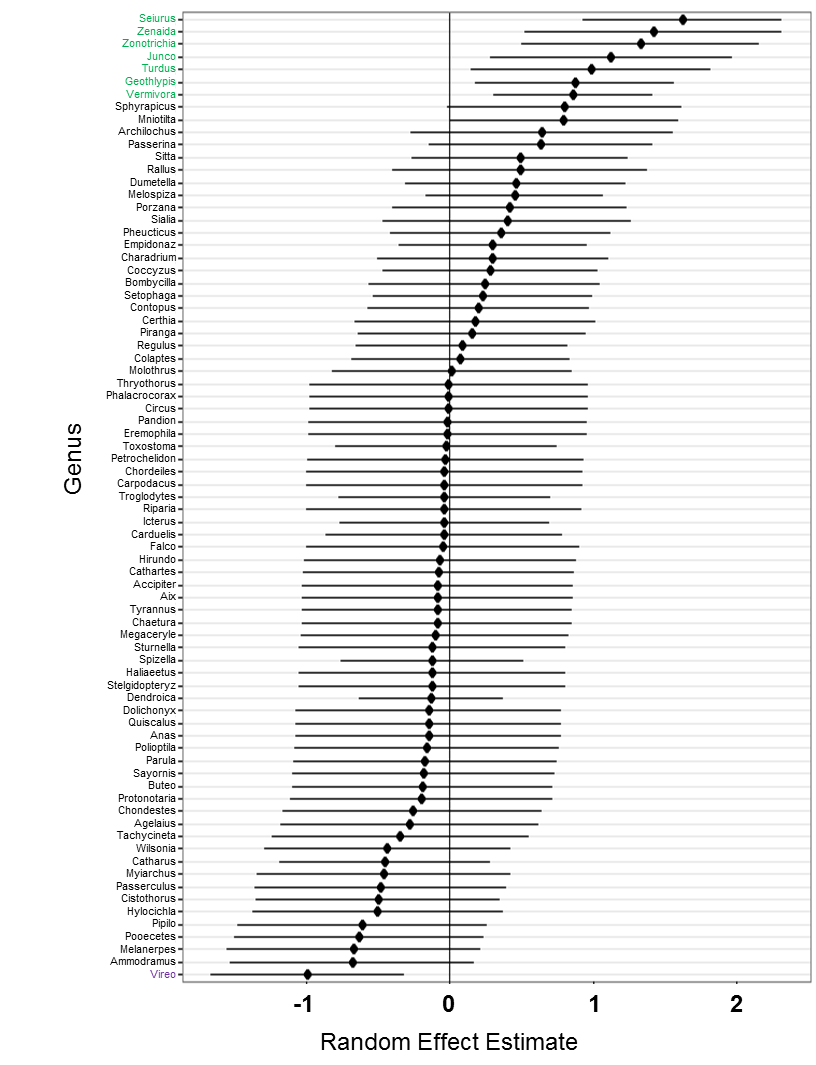

Supplement: S3 Fig — Only Vireo has a random effect estimate ± shrinkage estimate that is completely less than zero, indicating a superavoiding genus and is shown in purple. There are 7 genera that have random effect estimates ± shrinkage estimate that are completely greater than zero, indicating supercolliding genera: Seiurus, Zenaida, Zonotrichia, Junco, Turdus, Geothlypis, and Vermivora, and are shown in green. Note that 3 of these genera (Zenaida or Zenaida doves, Junco, and Turdus or thrushes) are single species genera with the species included in this analysis. Additionally, Seiurus or ovenbirds and Zonotrichia or American sparrows are 2-species genera with the species included in this analysis. (TIF) [file pone.0201558.s003.tif]
