# Supplementary material for: Bird-building collision risk: An assessment of the collision risk of birds with buildings by phylogeny and behavior using two citizen-science datasets
Source: PLoS One. 2018 Aug 9;13(8):e0201558. doi: 10.1371/journal.pone.0201558 (PMC6084936; doi:10.1371/journal.pone.0201558)
Supplement: S2 Table — Random effect estimates (REE) and shrinkage estimates (SE) for each species along with coefficient of abundance from top supported model (0.4349) and the correction factors used to center and scale the relative abundance values (adjusted abundance = 30.697*relative abundance– 0.6451) were used to calculate the change in abundance estimate necessary to result in a change in the classification of a species as supercollider, superavoider, or neither (ΔAbd). The classification of a species would change if REE ± SE for a supercollider or superavoider species was to shift to include 0 and for a species that was neither a supercollider nor superavoider, the classification would change if REE ± SE shifted to no longer include 0. ΔAbd was combined with the average relative abundance for each species to find the percent change in abundance necessary to change the classification of each species. Species are listed in order of increasing percent change. Species with common names in bold are classified as supercolliders or superavoiders. (DOCX) [file pone.0201558.s005.docx]

**S2 Table: Post-hoc test of robustness to detection probability errors.** Random effect estimates (REE) and shrinkage estimates (SE) for each species along with coefficient of abundance from top supported model (0.4349) and the correction factors used to center and scale the relative abundance values (adjusted abundance = 30.697*relative abundance – 0.6451) were used to calculate the change in abundance estimate necessary to result in a change in the classification of a species as supercollider, superavoider, or neither (ΔAbd). The classification of a species would change if REE ± SE for a supercollider or superavoider species was to shift to include 0 and for a species that was neither a supercollider nor superavoider, the classification would change if REE ± SE shifted to no longer include 0. ΔAbd was combined with the average relative abundance for each species to find the percent change in abundance necessary to change the classification of each species. Species are listed in order of increasing percent change. Species with common names in bold are classified as supercolliders or superavoiders.

| **Common Name** | **Scientific Name** | **REE** | **SE** | **REE - SE** | **REE + SE** | **Average Relative Abundance** | **Number of abundance records** | **ΔAbd** | **Percent Change** |
| --- | --- | --- | --- | --- | --- | --- | --- | --- | --- |
| Yellow-rumped Warbler | *Setophaga coronata* | -0.417 | 0.647 | -1.063 | 0.230 | 0.074 | 23 | 0.038 | 52% |
| Indigo Bunting | *Passerina cyanea* | 0.779 | 0.800 | -0.021 | 1.579 | 0.036 | 15 | 0.023 | 63% |
| Red-bellied Woodpecker | *Melanerpes carolinus* | -0.824 | 0.925 | -1.749 | 0.101 | 0.041 | 31 | 0.029 | 70% |
| Chipping Sparrow | *Spizella passerina* | 0.684 | 0.717 | -0.033 | 1.401 | 0.029 | 29 | 0.023 | 81% |
| American Goldfinch | *Spinus tristis* | -0.023 | 0.879 | -0.902 | 0.857 | 0.102 | 31 | 0.085 | 84% |
| American Robin | *Turdus migratorius* | 1.214 | 0.882 | 0.331 | 2.096 | 0.054 | 30 | 0.046 | 86% |
| Brown-headed Cowbird | *Molothrus ater* | 0.019 | 0.884 | -0.865 | 0.903 | 0.091 | 29 | 0.086 | 94% |
| Red-winged Blackbird | *Agelaius phoeniceus* | -0.345 | 0.973 | -1.318 | 0.628 | 0.063 | 28 | 0.068 | 108% |
| **White-breasted Nuthatch** | *Sitta carolinensis* | 1.073 | 0.823 | 0.250 | 1.896 | 0.036 | 30 | 0.040 | 110% |
| Song Sparrow | *Melospiza melodia* | -0.018 | 0.754 | -0.771 | 0.736 | 0.062 | 30 | 0.076 | 123% |
| American Redstart | *Setophaga ruticilla* | 0.285 | 0.786 | -0.501 | 1.070 | 0.044 | 16 | 0.059 | 134% |
| Red-eyed Vireo | *Vireo olivaceus* | 0.135 | 0.819 | -0.684 | 0.954 | 0.053 | 16 | 0.072 | 137% |
| Great Crested Flycatcher | *Myiarchus crinitus* | -0.569 | 0.939 | -1.507 | 0.370 | 0.035 | 18 | 0.049 | 139% |
| Ruby-crowned Kinglet | *Regulus calendula* | 0.520 | 0.802 | -0.281 | 1.322 | 0.030 | 20 | 0.042 | 140% |
| House Wren | *Troglodytes aedon* | 0.158 | 0.760 | -0.602 | 0.918 | 0.046 | 24 | 0.066 | 144% |
| Tree Swallow | *Tachycineta bicolor* | -0.426 | 0.966 | -1.393 | 0.540 | 0.039 | 29 | 0.061 | 156% |
| Field Sparrow | *Spizella pusilla* | -1.369 | 0.809 | -2.177 | -0.560 | 0.037 | 28 | 0.063 | 172% |
| Yellow-bellied Sapsucker | *Sphyrapicus varius* | 0.985 | 0.846 | 0.138 | 1.831 | 0.017 | 5 | 0.031 | 183% |
| Eastern Wood-Pewee | *Contopus virens* | 0.527 | 0.836 | -0.308 | 1.363 | 0.024 | 12 | 0.044 | 186% |
| Cedar Waxwing | *Bombycilla cedrorum* | 0.299 | 0.853 | -0.554 | 1.152 | 0.030 | 14 | 0.062 | 208% |
| Gray Catbird | *Dumetella carolinensis* | 0.566 | 0.788 | -0.222 | 1.355 | 0.015 | 19 | 0.038 | 253% |
| Nashville Warbler | *Oreothlypis ruficapilla* | 0.830 | 0.606 | 0.224 | 1.436 | 0.013 | 17 | 0.038 | 298% |
| Baltimore Oriole | *Icterus galbula* | -0.098 | 0.846 | -0.944 | 0.748 | 0.025 | 20 | 0.077 | 306% |
| **Yellow Warbler** | *Setophaga petechia* | 0.944 | 0.722 | 0.222 | 1.666 | 0.012 | 18 | 0.038 | 311% |
| Blue-gray Gnatcatcher | *Polioptila caerulea* | -0.197 | 1.007 | -1.204 | 0.809 | 0.024 | 22 | 0.082 | 347% |
| **Clay-colored Sparrow** | *Spizella pallida* | 0.534 | 0.776 | -0.242 | 1.309 | 0.010 | 20 | 0.039 | 380% |
| Vesper Sparrow | *Pooecetes gramineus* | -0.776 | 0.927 | -1.703 | 0.150 | 0.008 | 22 | 0.032 | 404% |
| Least Flycatcher | *Empidonax minimus* | 0.860 | 0.765 | 0.095 | 1.625 | 0.007 | 14 | 0.028 | 407% |
| Northern Flicker | *Colaptes auratus* | 0.091 | 0.778 | -0.687 | 0.869 | 0.018 | 30 | 0.072 | 407% |
| Rose-breasted Grosbeak | *Pheucticus ludovicianus* | 0.436 | 0.788 | -0.352 | 1.224 | 0.011 | 19 | 0.047 | 416% |
| **Tennessee Warbler** | *Oreothlypis peregrina* | 1.880 | 0.584 | 1.296 | 2.464 | 0.027 | 16 | 0.118 | 437% |
| Common Yellowthroat | *Geothlypis trichas* | 1.224 | 0.714 | 0.510 | 1.938 | 0.013 | 17 | 0.059 | 463% |
| Golden-crowned Kinglet | *Regulus satrapa* | -0.417 | 0.947 | -1.364 | 0.529 | 0.011 | 4 | 0.061 | 542% |
| Eastern Phoebe | *Sayornis phoebe* | -0.229 | 0.998 | -1.227 | 0.769 | 0.014 | 30 | 0.079 | 554% |
| **Eastern Bluebird** | *Sialia sialis* | 0.492 | 0.928 | -0.436 | 1.420 | 0.010 | 26 | 0.054 | 564% |
| Eastern Towhee | *Pipilo erythrophthalmus* | -0.752 | 0.927 | -1.680 | 0.175 | 0.006 | 21 | 0.034 | 568% |
| **Warbling Vireo** | *Vireo gilvus* | -0.476 | 0.935 | -1.411 | 0.459 | 0.010 | 14 | 0.055 | 571% |
| **Grasshopper Sparrow** | *Ammodramus savannarum* | -0.743 | 0.927 | -1.670 | 0.185 | 0.006 | 22 | 0.035 | 580% |
| White-throated Sparrow | *Zonotrichia albicollis* | 1.934 | 0.839 | 1.095 | 2.772 | 0.017 | 17 | 0.103 | 602% |
| Yellow-throated Vireo | *Vireo flavifrons* | -0.532 | 0.925 | -1.457 | 0.392 | 0.008 | 17 | 0.050 | 655% |
| Dark-eyed Junco | *Junco hyemalis* | 1.384 | 0.883 | 0.500 | 2.267 | 0.009 | 5 | 0.059 | 657% |
| Brown Thrasher | *Toxostoma rufum* | -0.032 | 0.801 | -0.832 | 0.769 | 0.010 | 25 | 0.079 | 819% |
| Palm Warbler | *Setophaga palmarum* | -0.267 | 0.759 | -1.026 | 0.491 | 0.007 | 15 | 0.058 | 838% |
| Common Grackle | *Quiscalus quiscula* | -0.184 | 1.010 | -1.194 | 0.827 | 0.010 | 24 | 0.083 | 846% |
| Blackpoll Warbler | *Setophaga striata* | 0.727 | 0.756 | -0.029 | 1.482 | 0.003 | 6 | 0.023 | 859% |
| Black-and-white Warbler | *Mniotilta varia* | 0.979 | 0.826 | 0.154 | 1.805 | 0.004 | 11 | 0.033 | 903% |
| Wood Thrush | *Hylocichla mustelina* | -0.621 | 0.935 | -1.556 | 0.315 | 0.005 | 14 | 0.045 | 970% |
| Mourning Dove | *Zenaida macroura* | 1.748 | 0.957 | 0.791 | 2.705 | 0.008 | 25 | 0.080 | 1,028% |
| Northern Rough-winged Swallow | *Stelgidopteryx serripennis* | -0.156 | 1.019 | -1.175 | 0.864 | 0.007 | 20 | 0.086 | 1,174% |
| Blue-winged Warbler | *Vermivora cyanoptera* | -0.792 | 0.865 | -1.657 | 0.073 | 0.002 | 6 | 0.026 | 1,205% |
| **Ovenbird** | *Seiurus aurocapilla* | 1.587 | 0.783 | 0.804 | 2.370 | 0.007 | 21 | 0.081 | 1,232% |
| Ruby-throated Hummingbird | *Archilochus colubris* | 0.788 | 0.986 | -0.198 | 1.774 | 0.003 | 9 | 0.036 | 1,234% |
| Bank Swallow | *Riparia riparia* | -0.047 | 1.061 | -1.108 | 1.014 | 0.008 | 5 | 0.097 | 1,242% |
| Eastern Kingbird | *Tyrannus tyrannus* | -0.109 | 1.035 | -1.144 | 0.927 | 0.007 | 13 | 0.090 | 1,310% |
| Chestnut-sided Warbler | *Setophaga pensylvanica* | 0.366 | 0.720 | -0.354 | 1.085 | 0.004 | 11 | 0.048 | 1,319% |
| Chimney Swift | *Chaetura pelagica* | -0.109 | 1.035 | -1.144 | 0.927 | 0.007 | 13 | 0.090 | 1,370% |
| Savannah Sparrow | *Passerculus sandwichensis* | -0.594 | 0.938 | -1.532 | 0.343 | 0.003 | 13 | 0.047 | 1,374% |
| **Black-throated Green Warbler** | *Setophaga virens* | -0.539 | 0.897 | -1.436 | 0.358 | 0.003 | 8 | 0.048 | 1,448% |
| Magnolia Warbler | *Setophaga magnolia* | -0.333 | 0.954 | -1.287 | 0.622 | 0.005 | 4 | 0.068 | 1,502% |
| Cliff Swallow | *Petrochelidon pyrrhonota* | -0.038 | 1.065 | -1.103 | 1.028 | 0.006 | 4 | 0.098 | 1,581% |
| Wood Duck | *Aix sponsa* | -0.108 | 1.038 | -1.146 | 0.931 | 0.005 | 1 | 0.091 | 1,711% |
| Orange-crowned Warbler | *Oreothlypis celata* | -0.362 | 0.805 | -1.167 | 0.443 | 0.003 | 7 | 0.054 | 1,748% |
| Winter Wren | *Troglodytes hiemalis* | -0.204 | 1.002 | -1.206 | 0.798 | 0.004 | 2 | 0.081 | 1,836% |
| Hermit Thrush | *Catharus guttatus* | -0.436 | 0.942 | -1.379 | 0.506 | 0.003 | 8 | 0.059 | 1,841% |
| Barn Swallow | *Hirundo rustica* | -0.085 | 1.045 | -1.130 | 0.959 | 0.005 | 10 | 0.093 | 1,896% |
| Lincoln's Sparrow | *Melospiza lincolnii* | 0.271 | 0.865 | -0.594 | 1.136 | 0.003 | 3 | 0.066 | 1,985% |
| Cooper's Hawk | *Accipiter cooperii* | -0.106 | 1.037 | -1.143 | 0.930 | 0.004 | 13 | 0.091 | 2,016% |
| Marsh Wren | *Cistothorus palustris* | -0.422 | 0.958 | -1.379 | 0.536 | 0.003 | 8 | 0.061 | 2,110% |
| Swamp Sparrow | *Melospiza georgiana* | 0.302 | 0.691 | -0.389 | 0.993 | 0.002 | 19 | 0.050 | 2,282% |
| Scarlet Tanager | *Piranga olivacea* | 0.190 | 0.831 | -0.641 | 1.020 | 0.003 | 11 | 0.069 | 2,300% |
| Alder Flycatcher | *Empidonax alnorum* | -0.439 | 0.936 | -1.375 | 0.496 | 0.003 | 4 | 0.058 | 2,328% |
| Eastern Meadowlark | *Sturnella magna* | -0.149 | 1.021 | -1.171 | 0.872 | 0.004 | 20 | 0.086 | 2,332% |
| Yellow-billed Cuckoo | *Coccyzus americanus* | 0.117 | 0.865 | -0.748 | 0.982 | 0.003 | 5 | 0.077 | 2,333% |
| Killdeer | *Charadrius vociferus* | 0.366 | 0.854 | -0.488 | 1.220 | 0.002 | 19 | 0.058 | 2,396% |
| Red-breasted Nuthatch | *Sitta canadensis* | -0.469 | 0.939 | -1.409 | 0.470 | 0.002 | 9 | 0.056 | 2,559% |
| **Orchard Oriole** | *Icterus spurius* | 0.050 | 0.853 | -0.802 | 0.903 | 0.003 | 8 | 0.081 | 2,616% |
| Swainson's Thrush | *Catharus ustulatus* | 0.151 | 0.863 | -0.713 | 1.014 | 0.003 | 10 | 0.074 | 2,657% |
| Northern Parula | *Setophaga americana* | -0.213 | 1.002 | -1.215 | 0.789 | 0.003 | 6 | 0.080 | 2,670% |
| Lark Sparrow | *Chondestes grammacus* | -0.321 | 0.976 | -1.296 | 0.655 | 0.003 | 11 | 0.070 | 2,696% |
| Blackburnian Warbler | *Setophaga fusca* | -0.326 | 0.956 | -1.282 | 0.630 | 0.002 | 4 | 0.068 | 2,728% |
| Mallard | *Anas platyrhynchos* | -0.184 | 1.013 | -1.197 | 0.828 | 0.003 | 2 | 0.083 | 2,866% |
| Northern Waterthrush | *Parkesia noveboracensis* | 0.410 | 0.906 | -0.496 | 1.315 | 0.002 | 5 | 0.058 | 2,910% |
| Bald Eagle | *Haliaeetus leucocephalus* | -0.154 | 1.020 | -1.174 | 0.866 | 0.003 | 21 | 0.086 | 2,962% |
| Brown Creeper | *Certhia americana* | 0.217 | 0.896 | -0.679 | 1.112 | 0.002 | 12 | 0.072 | 2,996% |
| Pine Warbler | *Setophaga pinus* | -0.101 | 1.038 | -1.139 | 0.937 | 0.003 | 1 | 0.091 | 3,040% |
| Virginia Rail | *Rallus limicola* | 0.601 | 0.958 | -0.357 | 1.559 | 0.002 | 1 | 0.048 | 3,180% |
| Unidentified Empidonax Flycatcher | *Empidonax (sp)* | 0.346 | 0.875 | -0.529 | 1.221 | 0.002 | 8 | 0.061 | 3,195% |
| Northern Harrier | *Circus cyaneus* | -0.010 | 1.079 | -1.089 | 1.070 | 0.003 | 1 | 0.101 | 3,265% |
| Blue-headed Vireo | *Vireo solitarius* | -0.348 | 0.962 | -1.310 | 0.615 | 0.002 | 10 | 0.067 | 3,355% |
| Henslow's Sparrow | *Ammodramus henslowii* | -0.096 | 1.041 | -1.137 | 0.944 | 0.003 | 4 | 0.092 | 3,396% |
| Sedge Wren | *Cistothorus platensis* | -0.195 | 1.008 | -1.203 | 0.813 | 0.002 | 3 | 0.082 | 3,413% |
| American Kestrel | *Falco sparverius* | -0.044 | 1.063 | -1.106 | 1.019 | 0.003 | 5 | 0.097 | 3,475% |
| Broad-winged Hawk | *Buteo platypterus* | -0.120 | 1.031 | -1.151 | 0.911 | 0.002 | 17 | 0.089 | 3,572% |
| Sora | *Porzana carolina* | 0.514 | 0.858 | -0.344 | 1.372 | 0.001 | 5 | 0.047 | 3,600% |
| Red-tailed Hawk | *Buteo jamaicensis* | -0.101 | 1.038 | -1.139 | 0.938 | 0.002 | 14 | 0.091 | 3,652% |
| Olive-sided Flycatcher | *Contopus cooperi* | -0.281 | 0.977 | -1.258 | 0.695 | 0.002 | 6 | 0.073 | 4,061% |
| Golden-winged Warbler | *Vermivora chrysoptera* | -0.499 | 0.924 | -1.423 | 0.426 | 0.001 | 7 | 0.053 | 4,069% |
| Black-billed Cuckoo | *Coccyzus erythropthalmus* | 0.232 | 0.877 | -0.645 | 1.108 | 0.002 | 4 | 0.069 | 4,331% |
| Belted Kingfisher | *Megaceryle alcyon* | -0.129 | 1.028 | -1.157 | 0.900 | 0.002 | 17 | 0.088 | 4,420% |
| **Pine Siskin** | *Spinus pinus* | -0.027 | 1.071 | -1.097 | 1.044 | 0.002 | 3 | 0.099 | 4,509% |
| **Turkey Vulture** | *Cathartes aura* | -0.096 | 1.040 | -1.137 | 0.944 | 0.002 | 12 | 0.092 | 4,826% |
| Mourning Warbler | *Geothlypis philadelphia* | -0.048 | 0.869 | -0.917 | 0.821 | 0.002 | 4 | 0.082 | 5,500% |
| Horned Lark | *Eremophila alpestris* | -0.018 | 1.075 | -1.093 | 1.056 | 0.002 | 2 | 0.100 | 5,567% |
| **Canada Warbler** | *Cardellina canadensis* | -0.394 | 0.962 | -1.356 | 0.569 | 0.001 | 7 | 0.064 | 5,782% |
| Willow Flycatcher | *Empidonax traillii* | -0.256 | 0.985 | -1.241 | 0.730 | 0.001 | 2 | 0.076 | 5,823% |
| Wilson's Warbler | *Cardellina pusilla* | -0.143 | 1.025 | -1.168 | 0.882 | 0.001 | 2 | 0.087 | 7,258% |
| Veery | *Catharus fuscescens* | -0.200 | 1.003 | -1.203 | 0.804 | 0.001 | 3 | 0.081 | 7,382% |
| Harris's Sparrow | *Zonotrichia querula* | -0.296 | 0.993 | -1.289 | 0.696 | 0.001 | 1 | 0.073 | 8,133% |
| Double-crested Cormorant | *Phalacrocorax auritus* | -0.009 | 1.080 | -1.089 | 1.070 | 0.001 | 1 | 0.101 | 8,433% |
| Prothonotary Warbler | *Protonotaria citrea* | -0.244 | 0.996 | -1.239 | 0.752 | 0.001 | 3 | 0.077 | 8,600% |
| Bobolink | *Dolichonyx oryzivorus* | -0.181 | 1.014 | -1.194 | 0.833 | 0.001 | 2 | 0.083 | 9,267% |
| Gray-cheeked Thrush | *Catharus minimus* | -0.075 | 1.050 | -1.124 | 0.975 | 0.001 | 1 | 0.094 | 9,400% |
| Purple Finch | *Haemorhous purpureus* | -0.046 | 1.062 | -1.108 | 1.016 | 0.001 | 1 | 0.097 | 10,789% |
| Yellow-bellied Flycatcher | *Empidonax flaviventris* | -0.141 | 1.025 | -1.166 | 0.884 | 0.001 | 1 | 0.087 | 10,900% |
| Osprey | *Pandion haliaetus* | -0.018 | 1.075 | -1.093 | 1.057 | 0.001 | 2 | 0.100 | 11,133% |
| Cape May Warbler | *Setophaga tigrina* | -0.118 | 1.029 | -1.147 | 0.911 | 0.001 | 3 | 0.089 | 11,163% |
| **Connecticut Warbler** | *Oporornis agilis* | -0.104 | 1.039 | -1.142 | 0.935 | 0.001 | 1 | 0.091 | 11,375% |
| Bay-breasted Warbler | *Setophaga castanea* | -0.099 | 1.039 | -1.138 | 0.940 | 0.001 | 1 | 0.091 | 11,425% |
| Common Nighthawk | *Chordeiles minor* | -0.046 | 1.062 | -1.108 | 1.016 | 0.001 | 1 | 0.097 | 12,138% |
| Merlin | *Falco columbarius* | -0.018 | 1.075 | -1.093 | 1.058 | 0.001 | 2 | 0.100 | 12,525% |
| Red-shouldered Hawk | *Buteo lineatus* | -0.015 | 1.076 | -1.092 | 1.061 | 0.001 | 2 | 0.101 | 12,563% |
| Carolina Wren | *Thryothorus ludovicianus* | -0.009 | 1.080 | -1.089 | 1.070 | 0.001 | 1 | 0.101 | 12,650% |
| Red-headed Woodpecker | *Melanerpes erythrocephalus* | -0.005 | 1.082 | -1.087 | 1.077 | 0.001 | 1 | 0.102 | 12,713% |
