# Supplementary material for: Bird-building collision risk: An assessment of the collision risk of birds with buildings by phylogeny and behavior using two citizen-science datasets
Source: PLoS One. 2018 Aug 9;13(8):e0201558. doi: 10.1371/journal.pone.0201558 (PMC6084936; doi:10.1371/journal.pone.0201558)
Supplement: S3 Table — Numbers represent the total number of individual birds recorded as colliding with a building during springs 2007 through 2010. Those species with no collisions are species that were observed in point counts but had no records of collisions during the study period. (DOCX) [file pone.0201558.s006.docx]

**S3 Table. Table of collisions of all permanent resident species removed from dataset prior to analysis.**

| **Common Name** | **Scientific Name** | **Number of Collisions** |
| --- | --- | --- |
| American Crow | *Corvus brachyrhynchos* | 0 |
| Barred Owl | *Strix varia* | 0 |
| Black-capped Chickadee | *Poecile atricapillus* | 3 |
| Blue Jay | *Cyanocitta cristata* | 6 |
| Downy Woodpecker | *Picoides pubescens* | 2 |
| European Starling | *Sturnus vulgaris* | 3 |
| Great Horned Owl | *Bubo virginianus* | 0 |
| Hairy Woodpecker | *Leuconotopicus villosus* | 0 |
| House Finch | *Haemorhous mexicanus* | 5 |
| House Sparrow | *Passer domesticus* | 5 |
| Northern Cardinal | *Cardinalis cardinalis* | 0 |
| Pileated Woodpecker | *Hylatomus pileatus* | 0 |
| Ring-necked Pheasant | *Phasianus colchicus* | 0 |
| Rock Pigeon | *Columba livia* | 8 |
| Wild Turkey | *Meleagris gallopavo* | 0 |

Numbers represent the total number of individual birds recorded as colliding with a building during springs 2007 through 2010. Those species with no collisions are species that were observed in point counts but had no records of collisions during the study period.
