# Supplementary material for: Bird-building collision risk: An assessment of the collision risk of birds with buildings by phylogeny and behavior using two citizen-science datasets
Source: PLoS One. 2018 Aug 9;13(8):e0201558. doi: 10.1371/journal.pone.0201558 (PMC6084936; doi:10.1371/journal.pone.0201558)
Supplement: S4 Table — Data removed from analysis due to uncertainty about the cause of the zero point-count detection. Table sorted by species common name and then by the first date of the week for each data point. Number of Collisions indicates the number of collisions by that species that were recorded by Project BirdSafe monitors during the week that started with the date for that row. 88 data points were removed with this restriction, representing 39 different species. Only 5 species were completely eliminated from the analysis with this restriction: American Woodcock (Scolopax minor), Eastern Whip-poor-will (Caprimulgus vociferus), Fox Sparrow (Passerella iliaca), Pied-billed Grebe (Podilymbus podiceps), and Rusty Blackbird (Euphagus carolinus). (DOCX) [file pone.0201558.s007.docx]

**S4 Table. Table of data points removed from analysis for having zero point-count detections in a week but a non-zero number of collisions in the same week.**

| **First Date of Week** | **Species Common Name** | **Number of Collisions** |
| --- | --- | --- |
| 4/4/2010 | American Robin | 1 |
| 4/22/2007 | American Woodcock | 1 |
| 5/6/2007 | American Woodcock | 3 |
| 3/29/2009 | American Woodcock | 1 |
| 4/5/2009 | American Woodcock | 1 |
| 4/11/2010 | American Woodcock | 1 |
| 5/20/2007 | Black-and-white Warbler | 1 |
| 5/27/2007 | Black-billed Cuckoo | 1 |
| 4/5/2009 | Brown-headed Cowbird | 1 |
| 5/4/2008 | Blackburnian Warbler | 1 |
| 5/23/2010 | Blackburnian Warbler | 1 |
| 4/6/2008 | Brown Creeper | 1 |
| 3/29/2009 | Brown Creeper | 1 |
| 4/5/2009 | Brown Creeper | 1 |
| 4/12/2009 | Brown Creeper | 4 |
| 3/28/2010 | Brown Creeper | 2 |
| 4/4/2010 | Brown Creeper | 1 |
| 4/11/2010 | Brown Creeper | 5 |
| 5/9/2010 | Brown Creeper | 2 |
| 5/9/2010 | Blue-winged Warbler | 1 |
| 5/13/2007 | Cape May Warbler | 1 |
| 4/1/2007 | Dark-eyed Junco | 1 |
| 3/30/2008 | Dark-eyed Junco | 2 |
| 4/6/2008 | Dark-eyed Junco | 3 |
| 4/13/2008 | Dark-eyed Junco | 2 |
| 3/29/2009 | Dark-eyed Junco | 1 |
| 4/5/2009 | Dark-eyed Junco | 2 |
| 4/19/2009 | Dark-eyed Junco | 1 |
| 3/28/2010 | Dark-eyed Junco | 7 |
| 4/4/2010 | Dark-eyed Junco | 7 |
| 4/11/2010 | Dark-eyed Junco | 2 |
| 4/25/2010 | Dark-eyed Junco | 1 |
| 4/13/2008 | Eastern Meadowlark | 1 |
| 5/11/2008 | Eastern Whip-poor-will | 1 |
| 4/22/2007 | Fox Sparrow | 1 |
| 5/16/2010 | Golden-winged Warbler | 1 |
| 4/22/2007 | Hermit Thrush | 1 |
| 4/18/2010 | Hermit Thrush | 1 |
| 4/13/2008 | Killdeer | 1 |
| 5/6/2007 | Lincoln's Sparrow | 1 |
| 5/11/2008 | Lincoln's Sparrow | 2 |
| 5/18/2008 | Lincoln's Sparrow | 1 |
| 5/4/2008 | Magnolia Warbler | 1 |
| 5/27/2007 | Mourning Dove | 1 |
| 4/5/2009 | Mourning Dove | 1 |
| 5/31/2009 | Mourning Warbler | 1 |
| 4/22/2007 | Nashville Warbler | 1 |
| 4/29/2007 | Nashville Warbler | 1 |
| 3/28/2010 | Northern Flicker | 1 |
| 5/6/2007 | Northern Waterthrush | 2 |
| 5/20/2007 | Northern Waterthrush | 1 |
| 4/19/2009 | Northern Waterthrush | 1 |
| 5/3/2009 | Northern Waterthrush | 1 |
| 5/10/2009 | Northern Waterthrush | 1 |
| 4/29/2007 | Orange-crowned Warbler | 1 |
| 5/31/2009 | Ovenbird | 1 |
| 4/22/2007 | Pied-billed Grebe | 1 |
| 5/20/2007 | Ruby-crowned Kinglet | 1 |
| 4/5/2009 | Ruby-crowned Kinglet | 1 |
| 4/22/2007 | Red-eyed Vireo | 1 |
| 4/12/2009 | Rusty Blackbird | 6 |
| 3/29/2009 | Red-winged Blackbird | 1 |
| 4/5/2009 | Red-winged Blackbird | 1 |
| 5/13/2007 | Sora | 1 |
| 5/20/2007 | Sora | 1 |
| 5/4/2008 | Sora | 1 |
| 5/11/2008 | Sora | 1 |
| 4/26/2009 | Sora | 1 |
| 5/10/2009 | Sora | 1 |
| 5/2/2010 | Sora | 1 |
| 4/29/2007 | Swamp Sparrow | 1 |
| 4/11/2010 | Swamp Sparrow | 1 |
| 4/12/2009 | Swainson's Thrush | 1 |
| 5/9/2010 | Swainson's Thrush | 1 |
| 4/19/2009 | Tennessee Warbler | 1 |
| 5/20/2007 | Unidentified Empidonax Flycatcher | 1 |
| 5/10/2009 | Virginia Rail | 1 |
| 5/9/2010 | Virginia Rail | 1 |
| 4/26/2009 | Winter Wren | 1 |
| 4/18/2010 | White-throated Sparrow | 1 |
| 5/16/2010 | White-throated Sparrow | 3 |
| 4/15/2007 | Yellow-bellied Sapsucker | 2 |
| 5/13/2007 | Yellow-bellied Sapsucker | 1 |
| 5/20/2007 | Yellow-bellied Sapsucker | 1 |
| 4/20/2008 | Yellow-bellied Sapsucker | 1 |
| 4/27/2008 | Yellow-bellied Sapsucker | 1 |
| 5/24/2009 | Yellow-bellied Sapsucker | 1 |
| 4/4/2010 | Yellow-bellied Sapsucker | 2 |

Data removed from analysis due to uncertainty about the cause of the zero point-count detection. Table sorted by species common name and then by the first date of the week for each data point. Number of Collisions indicates the number of collisions by that species that were recorded by Project BirdSafe monitors during the week that started with the date for that row. 88 data points were removed with this restriction, representing 39 different species. Only 5 species were completely eliminated from the analysis with this restriction: American Woodcock (*Scolopax minor*), Eastern Whip-poor-will (*Caprimulgus vociferus*), Fox Sparrow (*Passerella iliaca*), Pied-billed Grebe (*Podilymbus podiceps*), and Rusty Blackbird (*Euphagus carolinus*).
