# Supplementary material for: Bird-building collision risk: An assessment of the collision risk of birds with buildings by phylogeny and behavior using two citizen-science datasets
Source: PLoS One. 2018 Aug 9;13(8):e0201558. doi: 10.1371/journal.pone.0201558 (PMC6084936; doi:10.1371/journal.pone.0201558)
Supplement: S6 Table — For each species, the 3 random effects estimates for the Family, Genus, and Species were summed and the sum was then used as the exponent of e to calculate the Net IRR. In other words, for each species i, NetIRRi=eα^Family[Familyi]+α^Genus[Genusi]+α^Species[Speciesi]. IRR can be interpreted as the number of times it is more likely for that species to collide with a building than the model average. As a specific example, a White-throated Sparrow (Z. albicollis) is 26.12 times more likely to hit a building than the average nocturnal migrant with the same relative abundance during the same week of spring migration. (DOCX) [file pone.0201558.s009.docx]

**S6 Table. Table of the calculated Incidence-Rate Ratios (IRR) for all species.**

| **Species** | **Net Incidence-Rate Ratio** |
| --- | --- |
| White-throated Sparrow | 26.12 |
| Ovenbird | 24.76 |
| Mourning Dove | 23.73 |
| Tennessee Warbler | 15.46 |
| Dark-eyed Junco | 12.27 |
| American Robin | 9.02 |
| Common Yellowthroat | 8.13 |
| Northern Waterthrush | 7.63 |
| Yellow-bellied Sapsucker | 5.96 |
| Black-and-white Warbler | 5.90 |
| Nashville Warbler | 5.41 |
| White-breasted Nuthatch | 4.77 |
| Ruby-throated Hummingbird | 4.17 |
| Indigo Bunting | 4.10 |
| Least Flycatcher | 3.19 |
| Virginia Rail | 2.97 |
| Harris's Sparrow | 2.81 |
| Gray Catbird | 2.79 |
| Sora | 2.54 |
| Eastern Bluebird | 2.44 |
| Mourning Warbler | 2.28 |
| Yellow Warbler | 2.25 |
| Rose-breasted Grosbeak | 2.20 |
| Connecticut Warbler | 2.15 |
| Swamp Sparrow | 2.12 |
| Eastern Wood-Pewee | 2.07 |
| Lincoln's Sparrow | 2.06 |
| Killdeer | 1.94 |
| Unidentified Empidonax Flycatcher | 1.91 |
| Ruby-crowned Kinglet | 1.83 |
| Blackpoll Warbler | 1.81 |
| Chipping Sparrow | 1.75 |
| Cedar Waxwing | 1.72 |
| American Redstart | 1.67 |
| Black-billed Cuckoo | 1.67 |
| Orange-crowned Warbler | 1.64 |
| Song Sparrow | 1.54 |
| Clay-colored Sparrow | 1.51 |
| Yellow-billed Cuckoo | 1.49 |
| Brown Creeper | 1.48 |
| Golden-winged Warbler | 1.43 |
| Scarlet Tanager | 1.41 |
| Chestnut-sided Warbler | 1.26 |
| Northern Flicker | 1.18 |
| Yellow-bellied Flycatcher | 1.17 |
| House Wren | 1.13 |
| Blue-winged Warbler | 1.07 |
| Willow Flycatcher | 1.05 |
| Brown-headed Cowbird | 1.03 |
| Red-breasted Nuthatch | 1.02 |
| Orchard Oriole | 1.01 |
| Carolina Wren | 0.98 |
| Double-crested Cormorant | 0.98 |
| Northern Harrier | 0.98 |
| Osprey | 0.97 |
| Horned Lark | 0.97 |
| Brown Thrasher | 0.94 |
| American Goldfinch | 0.94 |
| Pine Siskin | 0.94 |
| Merlin | 0.93 |
| Cliff Swallow | 0.93 |
| Olive-sided Flycatcher | 0.92 |
| Common Nighthawk | 0.92 |
| Purple Finch | 0.92 |
| Bank Swallow | 0.92 |
| American Kestrel | 0.91 |
| Baltimore Oriole | 0.87 |
| Alder Flycatcher | 0.87 |
| Barn Swallow | 0.86 |
| Turkey Vulture | 0.84 |
| Cooper's Hawk | 0.83 |
| Wood Duck | 0.82 |
| Eastern Kingbird | 0.82 |
| Chimney Swift | 0.82 |
| Red-shouldered Hawk | 0.81 |
| Bay-breasted Warbler | 0.79 |
| Belted Kingfisher | 0.79 |
| Pine Warbler | 0.79 |
| Winter Wren | 0.78 |
| Cape May Warbler | 0.78 |
| Eastern Meadowlark | 0.76 |
| Bald Eagle | 0.76 |
| Northern Rough-winged Swallow | 0.75 |
| Red-tailed Hawk | 0.75 |
| Swainson's Thrush | 0.74 |
| Broad-winged Hawk | 0.73 |
| Bobolink | 0.72 |
| Common Grackle | 0.72 |
| Golden-crowned Kinglet | 0.72 |
| Mallard | 0.72 |
| Blue-gray Gnatcatcher | 0.70 |
| Northern Parula | 0.68 |
| Palm Warbler | 0.67 |
| Eastern Phoebe | 0.66 |
| Prothonotary Warbler | 0.64 |
| Blackburnian Warbler | 0.63 |
| Magnolia Warbler | 0.63 |
| Gray-cheeked Thrush | 0.59 |
| Yellow-rumped Warbler | 0.58 |
| Wilson's Warbler | 0.56 |
| Lark Sparrow | 0.56 |
| Red-winged Blackbird | 0.54 |
| Veery | 0.52 |
| Black-throated Green Warbler | 0.51 |
| Red-headed Woodpecker | 0.51 |
| Sedge Wren | 0.50 |
| Tree Swallow | 0.46 |
| Henslow's Sparrow | 0.46 |
| Canada Warbler | 0.44 |
| Red-eyed Vireo | 0.42 |
| Hermit Thrush | 0.41 |
| Marsh Wren | 0.40 |
| Great Crested Flycatcher | 0.36 |
| Savannah Sparrow | 0.34 |
| Wood Thrush | 0.32 |
| Blue-headed Vireo | 0.26 |
| Eastern Towhee | 0.26 |
| Vesper Sparrow | 0.24 |
| Grasshopper Sparrow | 0.24 |
| Warbling Vireo | 0.23 |
| Field Sparrow | 0.23 |
| Red-bellied Woodpecker | 0.22 |
| Yellow-throated Vireo | 0.22 |

For each species, the 3 random effects estimates for the Family, Genus, and Species were summed and the sum was then used as the exponent of *e* to calculate the Net IRR. In other words, for each species *i*, ${Net IRR}_{i}= e^{\hat{\alpha}_{Family}\left[ {Family}_{i} \right] + \hat{\alpha}_{Genus}\left[ {Genus}_{i} \right]+ \hat{\alpha}_{Species}[{Species}_{i}]}$. IRR can be interpreted as the number of times it is more likely for that species to collide with a building than the model average. As a specific example, a White-throated Sparrow (*Z. albicollis*) is 26.12 times more likely to hit a building than the average nocturnal migrant with the same relative abundance during the same week of spring migration.
