# Supplementary material for: Bird-building collision risk: An assessment of the collision risk of birds with buildings by phylogeny and behavior using two citizen-science datasets
Source: PLoS One. 2018 Aug 9;13(8):e0201558. doi: 10.1371/journal.pone.0201558 (PMC6084936; doi:10.1371/journal.pone.0201558)
Supplement: S1 Appendix — (DOCX) [file pone.0201558.s011.docx]

# S1 Appendix: Post-hoc assessment of random effect estimate robustness to detection probability variability

To assess the strength of the analysis to errors in variable detection probability, I did a post-hoc analysis to test for the change in relative abundance necessary to result in a change in the classification of each species as supercollider, superavoider, or neither. By calculating the percent change necessary to alter the classification of a species, I found a measure of the amount of error (potentially caused by unaccounted for detection probability differences) that would be necessary to change the outcomes of the analysis. To do this post-hoc analysis, I found the minimum change in the random effect estimate (REE) that would be required to shift the REE ± the shrinkage estimate (SE) to include zero (in the case of supercolliders and superavoiders) or exclude zero (in the case of species that were neither supercolliders nor superavoiders). I took this value for each species (*x*) and found the equivalent change in adjusted abundance (i.e. divided the former by the coefficient estimate of abundance from the top model) and then converted those values to actual relative abundance (reversing the centering around the mean and scaling by standard deviation). The resulting value I took to be the change in relative abundance that would be necessary to change the classification of a species (I called this ΔAbd). This calculation was $\Delta Abd=\frac{(\frac{x}{0.4349} + 0.6451)}{30.697}$, where *x* equaled the change in REE necessary to change a species’ classification. For supercolliders, *x* equaled REE + SE; for superavoiders, *x* equaled RRR – SE; and for species that were neither supercolliders nor superavoiders, *x* equaled either REE + SE or REE – SE, whichever of those two values had the minimum absolute value (i.e. was closer to zero).

I then calculated the average observed relative abundance for each of these 13 species. Finally, I divided the ΔAbd by the average observed abundance for each species, to find the percent change in relative abundance necessary to change the classification of these species (S2 Table). The lowest percent change necessary that I found was 52% change in average relative abundance (for Yellow-rumped Warbler, *Setophaga coronata*). The average percent change necessary across all species was 2968%. This post hoc analysis assumes that errors in detection probability would only affect the REE and not the SE (a measure of variance of REE) outputs from the model, which is reasonable if unaccounted for detection probability were to only affect the values and not the sample size for each species, which is more likely to affect the variance of estimates of REE. Based on this rough post-hoc assessment, it does not seem likely that such drastic changes in relative abundance would be caused by unaccounted for biases in detection probability.
